# SLC26A3/NHERF2-IκB/NFκB/p65 feedback loop suppresses tumorigenesis and metastasis in colorectal cancer

**DOI:** 10.1038/s41389-023-00488-w

**Published:** 2023-08-12

**Authors:** Chunlin Lin, Penghang Lin, Huayan Lin, Hengxin Yao, Songyi Liu, Ruofan He, Hui Chen, Zuhong Teng, Robert M. Hoffman, Jianxin Ye, Guangwei Zhu

**Affiliations:** 1 Department of Gastrointestinal Surgery 2 Section, Institute of Abdominal Surgery, Key Laboratory of accurate diagnosis and treatment of cancer, The First Hospital Affiliated to Fujian Medical University, Fuzhou, 350005 China; 2 Key Laboratory of Ministry of Education for Gastrointestinal Cancer, Fujian Medical University, Fuzhou, 350000 China; 3 National Regional Medical Center, Binhai Campus of the First Affiliated Hospital, Fujian Medical University, Fuzhou, China; 4 AntiCancer, Inc, San Diego, CA USA; 5 Department of Surgery, University of California, San Diego, CA USA

**Keywords:** Colorectal cancer, Ubiquitylation

## Abstract

Colorectal cancer (CRC) is a formidable disease due to the intricate mechanisms that drive its proliferation and metastasis. Despite significant progress in cancer research, the integration of these mechanisms that influence cancer cell behavior remains elusive. Therefore, it is imperative to comprehensively elucidate the underlying mechanisms driving CRC proliferation and metastasis. In this study, we reported a novel role of SLC26A3 in suppressing CRC progression. We found that SLC26A3 expression was downregulated in CRC, which was proportionally correlated with survival. Our in vivo and in vitro experiments demonstrated that up-regulation of SLC26A3 inhibited CRC proliferation and metastasis, while down-regulation of SLC26A3 promoted CRC progression by modulating the expression level of IκB. Furthermore, we identified NHERF2 as a novel interacting protein of SLC26A3 responsible for stabilizing the IκB protein and removing ubiquitination modification. Mechanistically, SLC26A3 augmented the interaction between NHERF2 and IκB, subsequently reducing its degradation. This process inhibited the dissociation of p65 from the IκB/p65/p50 complex and reduced the translocation of p65 from the cytoplasm to the nucleus. Moreover, our investigation revealed that NF-κB/p65 directly bound to the promoter of SLC26A3, leading to a decline in its mRNA expression. Thus, SLC26A3 impeded the nuclear translocation of NF-κB/p65, enhancing the transcription of SLC26A3 and establishing a positive regulatory feedback loop in CRC cells. Collectively, these results suggest that a SLC26A3/NHERF2-IκB/NF-κB/p65 signaling loop suppresses proliferation and metastasis in CRC cells. These findings propose a novel SLC26A3-driven signaling loop that regulates proliferation and metastasis in CRC, providing promising therapeutic interventions and prognostic targets for the management of CRC.

CRC is the third most common type of cancer worldwide, with 1,360,600 new cases and 693,900 deaths reported in 2012. The incidence of CRC is gradually increasing due to various factors such as tobacco use, overweight, and poor dietary habits [1]. Furthermore, the 5-year survival rate for early-stage and localized CRC patients was over 90%, while for those with advanced stages or distant metastasis, it was less than 10%, despite significant advancements in therapy and surgical techniques [2, 3]. Hence, there is an urgent need to identify biomarker molecules that can aid in early diagnosis and treatment to decrease the mortality associated with CRC. Therefore, it is crucial to identify key molecular targets involved in CRC pathogenesis to improve the prognosis of patients.

The mechanisms underlying CRC prognosis, including metastasis, remain poorly understood. SLC26A3 is a cell membrane transporter and a member of the solute carrier transporters (SLCs) family. As an anion exchanger, SLC26A3 plays multiple important roles in the intestine. It is involved in the cellular response to Cl^-^ transmembrane absorption and HCO_3_^−^ secretion [4, 5], while maintaining the integrity and stability of the intestinal epithelial barrier [6, 7]. Currently, research on SLC26A3 primarily focuses on its significant role in congenital chloride diarrhea and inflammatory bowel disease [8]. Ding et al. reported that SLC26A3 might reverse the TNF-α-induced damage of the epithelial barrier by stabilizing tight junction proteins [9]. Furthermore, Chatterjee et al. demonstrated that the expression level of SLC26A3 is regulated by CDX2, affecting inflammatory bowel disease-associated diarrhea [10]. Importantly, SLC26A3 is down-regulated in CRC tissues [11, 12], and we identified it as a significantly down-regulated protein in human CRC that may influence tumor progression through the NF-κB signaling pathway.

The NF-κB signaling pathway has been widely implicated in cancer progression, including cell proliferation, apoptosis, necrosis, and metastasis. This is attributed to the critical role of NF-κB transcription factors in regulating multiple downstream genes [13, 14]. However, the NF-κB complex (p65/p50 subunits) is primarily located in the cytoplasm by binding to the inhibitory protein IκB. Upon degradation of IκB protein, p65 and p50 dissociate from the complex and translocate to the nucleus, where they regulate the mRNA expression level of downstream target genes [15]. Indeed, regulation of the NF-κB complex’s nuclear translocation through changes in the protein expression level of IκB plays a pivotal role in the expression of various related genes [16]. Therefore, this study aimed to verify the hypothesis that SLC26A3 inhibits malignant behaviors of CRC cells through the NF-κB signaling pathway.

Despite significant research findings, little is known about the function of SLC26A3 in human colorectal cancer. Therefore, further studies are necessary to investigate the effect of SLC26A3 on the progression of colorectal cancer.

NHERF2 (Na^+^/H^+^ exchanger regulatory factor 2) is a protein with two PDZ domains that act as protein–protein interacting sequences and scaffold proteins [17–19], interacting with numerous ion transporters and transmembrane proteins such as CFTR and LPA2 [20, 21]. Based on these specific functional domains, multiple studies have indicated that NHERF2 plays an important role in various diseases. Previous research reports that NHERF2 protein recruits PTEN to PDGFR, limiting the activation of PI3K. Moreover, it may decrease the expression of c-Myc or cyclin D1, inhibiting the proliferation of endothelial cells [22, 23]. However, the role of NHERF2 in the inhibition of CRC and its underlying mechanisms remain unknown.

This study aimed to evaluate the functional and clinical characteristics of SLC26A3 in CRC while investigating the specific mechanism by which SLC26A3 regulates IκB resulting in NF-κB/p65 nuclear translocation mediated by NHERF2. Additionally, we delved deeply into how NF-κB/p65 regulates the expression level of SLC26A3. The findings revealed the existence of a regulatory network consisting of a feedback loop between SLC26A3/NHERF2-IκB/NF-κB/p65, which influences the prognosis of CRC.

## Methods

### CRC patients and clinical samples

Clinical samples from CRC patients were collected for this study (Supplementary Table S1). A total of 100 pairs of CRC tissues and corresponding adjacent normal tissues were obtained from The First Affiliated Hospital of Fujian Medical University (Fuzhou, China) to verify SLC26A3’s expression levels using Western blotting and immunohistochemical (IHC) analysis.

All patients underwent surgery between March 2011 and March 2016. For IHC specimens, samples were fixed in formalin and embedded in paraffin. Detailed clinicopathological data, including age, gender, clinical stage, depth of tumor invasion, lymph node metastasis, distant metastasis, tumor differentiation, lymphovascular invasion, and nerve invasion were collected from hospital medical records. Patients were followed up until March 2020.

The median IHC score for SLC26A3 protein in patient samples was 1.75 points. Based on their IHC scores, all patients were divided into two groups: the high expression group (scores above 1.75), and the low expression group (scores below 1.75).

### Immunohistochemistry and scoring

The IHC staining analysis and blind scoring of SLC26A3 expression in clinical samples were performed following previously described methods [24]. Two pathologists scored all samples, and the maximum IHC score attainable was 12.

### Cell lines and cell culture

Human cell lines (Caco2, HCT116, sw480, and HEK293T) were obtained from the American Type Culture Collection (ATCC, http://www.atcc.org/). All cell lines were maintained in Dulbecco’s modified Eagle’s medium (DMEM) supplemented with 10% fetal bovine serum (FBS) and incubated in 5% CO2 at 37 °C.

### RNA isolation and qPCR

Total RNA was extracted from cell lines using Trizol regent (Life Technologies) and subsequently transcribed into cDNA using the Evo M-MLV RT Kit for qPCR (AG11707, Accurate Biotechnology, Hunan, Co., Ltd). The expression levels of target RNAs were detected using the SYBR Green Premix Pro Taq HS qPCR Kit (AG11701, Accurate Biotechnology, Hunan, Co., Ltd). GAPDH was used as an endogenous control to normalize gene expression, and fold change was measured via the 2^-ΔΔCt^ method. Table S2 lists all primers used in this study.

### Plasmids and transfection

The open reading frames of human SLC26A3, NHERF2, and p65 were inserted into the eukaryotic expression vector GFP-pCDH (System Biosciences, Inc.). The recombinant plasmid pCDH-SLC26A3, as well as lentivirus plasmids psPAX2 and pMD2.G (Genechem Inc, Shanghai, China), were co-transfected into HEK293T cells at 60% confluence using Lipofectamine 3.0 (Invitrogen) according to the manufacturer’s instructions for 48 h. HCT116 and Caco2 cells were transfected with lentivirus and subsequently selected with puromycin at 1 μg/mL (Invitrogen) for two weeks. Similarly, pCDH-NHERF2 and pCDH-p65 were treated as pCDH-SLC26A3.

### Cell proliferation assay

Cell proliferation was measured using a Cell Counting Kit-8 (CCK-8; Dojindo, Japan). A density of 1000 cells per well were seeded into 96-well plates and incubated at 5% CO_2_, 37 °C for 24, 48, 72, or 96 h. Serum-free DMEM with 10% CCK-8 was added to each well for 2 h, and absorbance was detected at 450 nm.

### Colony formation assay

Cells were seeded at a density of 800 cells per well in 6-well dishes and cultured at 37 °C with 5% CO_2_ for two weeks. Colonies were fixed in methanol and stained with crystal violet for 10 min, after which colonies containing 50 or more cells were counted.

### Cell migration and invasion assay

Cell migration assays were carried out using Transwell inserts (BD Biosciences, San Jose, CA, USA). The bottom chambers were filled with DMEM medium supplemented with 20% fetal bovine serum (FBS), while cell suspensions in serum-free DMEM medium were seeded into the upper chamber for 48 h. For cell invasion assays, Transwell inserts coated with Matrigel were used. DMEM medium containing 20% FBS was added to the bottom chambers, while a cell suspension of 2 × 10^5/0.3 mL in serum-free DMEM medium was seeded into the upper chamber for 48 h. After incubation, the chambers were fixed in methanol and stained with crystal violet (10 min per step). Cells that did not migrate through the upper chambers were removed using a cotton swab, and cells on the bottom of the upper chamber were counted under an inverted phase contrast microscope (Olympus).

### Dual-luciferase reporter assay

The SLC26A3 promoter Firefly luciferase reporter plasmid and pRL-TK plasmid were co-transfected with pCDNA3.1-p65 and the pCDNA3.1 empty plasmid, respectively. The Renilla luciferase expression plasmid pRL-TK was utilized as an internal control for normalization purposes, while the empty plasmid pCDNA3.1 served as a negative control. After 48 h of transfection, cell lysates were obtained using the Dual-luciferase Reporter Assay kit (Vazyme). The fluorescence intensity was measured using the Orion II Microplate Luminometer from Berthold Detection Systems. Each experiment was performed in triplicate, and the resulting data were expressed as mean ± standard deviation (SD) of three independent experiments.

### Chromatin immunoprecipitation assay (Chip)

The ChIP experiment was performed using the Enzymatic Chromatin IP Kit (9002 S, Cell Signaling Technology) according to the manufacturer’s protocol. Briefly, CRC cells transfected with the pCDNA3.1-p65 plasmid were treated with 1% formaldehyde to crosslink DNA and proteins; subsequently, the chromatin was sonicated and immunoprecipitated with 5 μg of anti-p65 antibody (8242 S, Cell Signaling Technology) at 4 °C overnight, while rabbit IgG served as negative control. After DNA extraction, PCR was performed to test the bound target DNA fragments. The amplified region consisted of −925 to −725 bp of the SLC26A3 promoter. Finally, the PCR products were visualized on a 1.8% agarose gel and stained with a nucleic acid-specific stain.

### Animal studies

All animal experiments were carried out by the "Guidelines for the Care and Use of Laboratory Animals of the National Institutes of Health", and animal experiments were carried out in the Animal Experiment Center of Fujian Medical University and approved by the University Ethics Committee.

We have generated three distinct mouse models encompassing subcutaneous and orthotopic model, as well as a lung metastasis model. For each of these models, we have designated four experimental groups: SLC26A3 overexpression, SLC26A3 knockdown, and their respective negative control groups.

Subcutaneous and Orthotopic Models: four group cells (1 × 10^6/200 μL PBS) were subcutaneously injected into 5-week-old male BALB/c nude mice (5 mice per group). Tumor length (L) and width (W) were measured every four days, and tumor volume was calculated as follows: Volume (mm^3^) = 0.5 * (W^2^ * L). Mice were sacrificed at 4–5 weeks after injection, and the tumors were removed, weighed, photographed, and collected for further analysis. For orthotopic models, tumors from the aforementioned subcutaneous models were minced into 2–3 mm^3 cubes and transplanted into the colon of BALB/c mice (5 mice per group). Six weeks after transplantation, mesenteries of 5 mice in each group were excised, and the number of swollen lymph nodes on the mesenteric surfaces was counted.

Lung Metastasis Models: four group cells (1 × 10^6/100 μL PBS) were intravenously injected via the tail vein of 5-week-old male BALB/c nude mice (5 mice per group). Six weeks after injection, the mice were euthanized, and the number of metastases formed on the lung surfaces were counted. Lung tissues were collected, fixed in paraffin, and prepared for further analysis.

### Immunofluorescent staining

We have conducted several immunofluorescence experiments to investigate changes in endogenous protein expression and the co-localization of exogenous proteins. As an example, the cell immunofluorescence co-localization method was used to examine SLC26A3 and NHERF2 exogenous protein interactions.

HCT116 and Caco2 cells were seeded on 3.5-cm dishes and transiently co-transfected with pCDNA3.1-Flag-SLC26A3 and pCDNA3.1-Myc-NHERF2 plasmids for 48 h. Subsequently, the cells were fixed in 4% precooled paraformaldehyde and permeabilized with 0.1% Triton X-100 at room temperature for 10 min. Following blocking with 10% TBS for 30 min, the cells were incubated overnight at 4 °C with antibodies against Flag-Tag (1:800; CST) and Myc-Tag (1:800; CST). The next day, Alexa Flour 488-conjugated goat anti-mouse secondary antibody (1:200; Abcam) and Alexa Flour 647-conjugated goat anti-rabbit secondary antibody (1:200; Abcam) were used to label the cells. Nuclei were stained with DAPI (Abcam), and the cells were observed using a laser scanning confocal microscope TCS SP8 (Leica Microsystems, Wetzlar, Germany).

### Co-immunoprecipitation and mass spectrometric analysis

The Flag-tagged SLC26A3 expression plasmid or the empty vectors were transfected into HEK293T cells. Co-immunoprecipitation was performed using anti-Flag antibody to identify SLC26A3 interaction proteins. The resulting protein complex was separated by SDS-PAGE for 30 min and stained with Coomassie blue (Beyotime Biotechnology). Subsequently, all gel lanes that were stained with Coomassie blue were excised for mass spectrometry analysis.

### Statistical analysis

GraphPad Prism Software 8.0 and SPSS 22.0 were utilized for statistical analysis. The data are expressed as mean ± Standard Deviation (SD) of at least three independent experiments. Student’s *t* test was employed to compare two independent groups, while the Pearson χ2 test was used to assess the correlation between SLC26A3 expression levels and clinicopathological parameters in CRC patients. Survival curves were generated using Kaplan–Meier analysis and further analyzed using the log-rank test. Statistical differences were considered significant under *p ≤ 0.05, **p < 0.01, ***p < 0.001.

## Results

### SLC26A3 is downregulated in CRC and proportionally correlated with survival

**Fig. 1:**
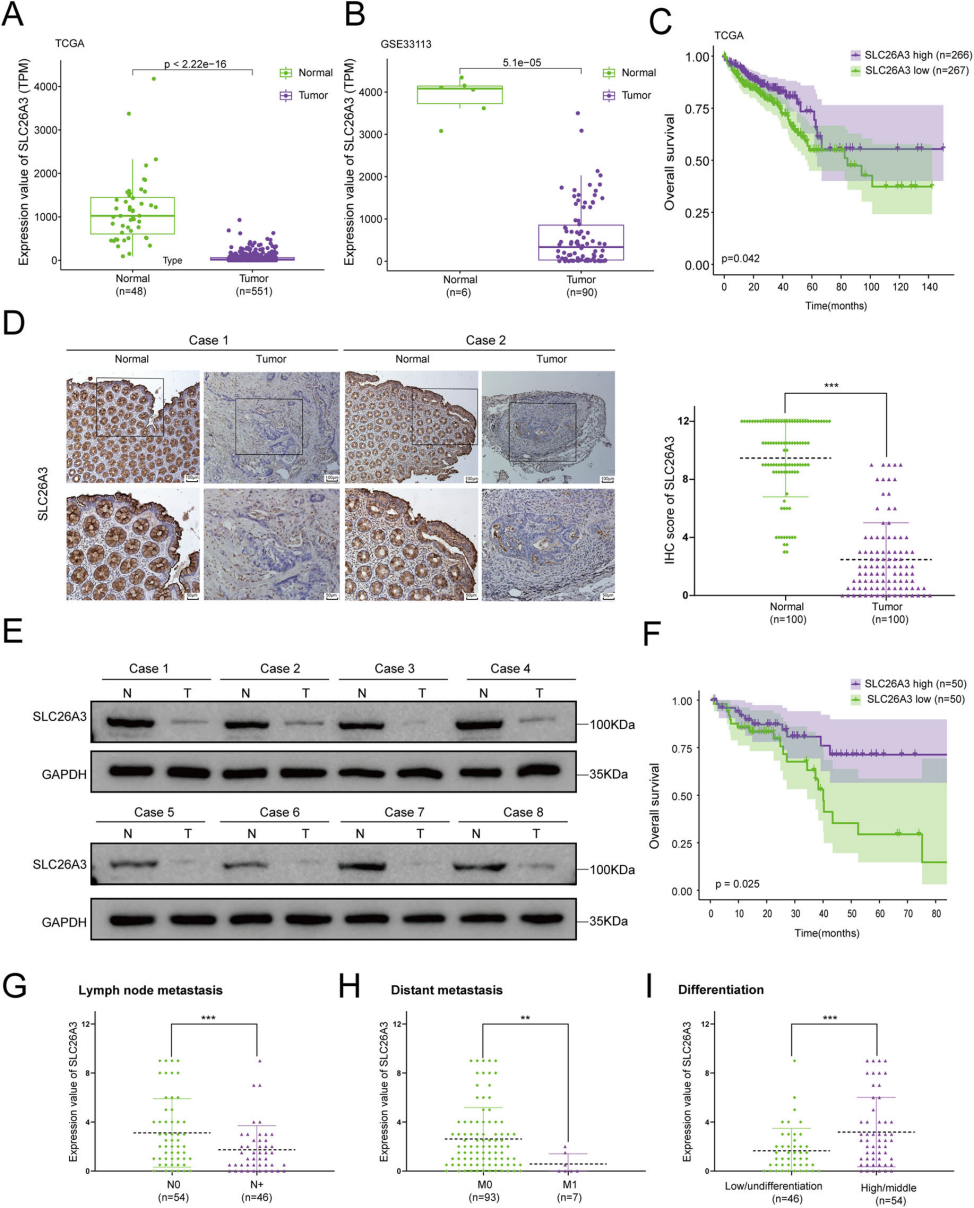
Reduced SLC26A3 expression is found in CRC and correlates to reduce the survival of CRC patients. SLC26A3 mRNA levels were lower in CRC tissues according to the TCGA (**A**) and GEO (**B**, GSE33113) databases. **C** Kaplan–Meier curves of overall survival in CRC according to expression of SLC26A3 in TCGA database. According to the SLC26A3 expression value, the patients were divided into two groups, and those greater than the mean value were the high expression group, and the rest are the low expression group, and finally combine the patient’s clinical data for survival analysis. **D** Representative immunohistochemistry staining images of SLC26A3 in CRC tissues and normal tissues (*n* = 100). Quantitative analysis was showed in the graphs. **E** Representative images of SLC26A3 protein levels in CRC tumor and normal tissues by Western blotting assays. **F** Kaplan–Meier curves of overall survival in CRC according to expression of SLC26A3 in clinical CRC patients (*n* = 100). The median of the IHC score of SLC26A3 protein in patient samples is 1.75 points. All patients were divided into two groups according to the result of their IHC score. The higher than 1.75 points are regarded as the high expression group (*n* = 50), and the lower expression group is below 1.75 (*n* = 50). **G** Lower expression of SLC26A3 was associated with increased lymph node metastasis in clinical CRC patients. **H** Lower expression of SLC26A3 was associated with increased distant metastasis in clinical CRC patients. **I** Lower expression of SLC26A3 correlated with worse pathological type in clinical CRC patient tissues.

To investigate the role of SLC26A3 in CRC, we initially examined its expression in CRC tissues. Based on analysis of the TCGA and GEO (GSE33113) databases, the mRNA levels of SLC26A3 were found to be lower in CRC tissues compared to normal tissues (Fig. 1A, B). Furthermore, based on their SLC26A3 expression values, patients were divided into two groups: high-expression group (expression value greater than mean value) and low-expression group. Finally, patient clinical data were combined for survival analysis. Overall survival of CRC patients was calculated using the Kaplan–Meier method. The results indicated that patients with high SLC26A3 expression exhibited a significantly higher overall survival rate compared to those with low expression in the TCGA database (Fig. 1C).

SLC26A3 protein expression levels in clinical patient samples were further evaluated using IHC staining and western blotting. The results showed that the SLC26A3 protein expression level was approximately four times higher in normal tissues than in cancerous tissues, consistently across both techniques (Fig. 1D, E and Fig. S1). The median IHC score for SLC26A3 protein in patient samples was 1.75 points. Patients were divided into two groups based on their IHC scores, with scores greater than 1.75 points classified as the high-expression group (*n* = 50), and those with lower scores as the low-expression group (*n* = 50). To determine the relationship between SLC26A3 expression levels and clinicopathological features in CRC cancer, we analyzed data collected from clinical CRC patients. The results suggested that low expression levels of SLC26A3 were associated with poor survival rates in CRC patients (Fig. 1F). Moreover, the data also indicated that low expression of SLC26A3 was related to higher metastasis rates, higher lymph node metastasis rates, and low or undifferentiated histopathology [25] (Fig. 1G–I and Table S1). These findings demonstrate that SLC26A3 is reduced in CRC tissues, and downregulation of SLC26A3 expression is associated with CRC prognosis, metastasis, and differentiation, highlighting SLC26A3 as a potential target for intervention in CRC.

### SLC26A3 inhibited the malignant behaviors of CRC cells

**Fig. 2:**
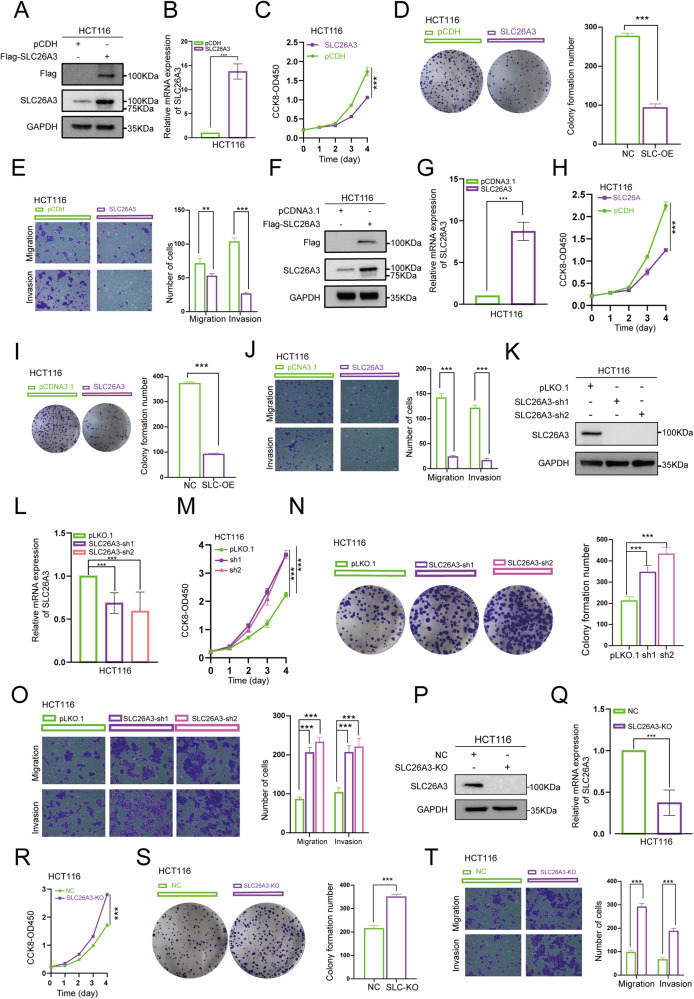
SLC26A3 inhibited the malignant behaviors of CRC cells. **A**–**E** HCT116 cells were stably transfected with either Flag-SLC26A3 or a mock control plasmid. Western blotting and qPCR were performed to detect the expression levels of SLC26A3 (**A**, **B**). The transcript levels of SLC26A3 were normalized to the expression of GAPDH, and negative control cells were used as the baseline value of 1. The transfected cells were further assessed for their proliferation ability using CCK-8 assay (**C**) and colony formation assay (**D**), as well as migration and invasion ability using Transwell assay (**E**). The corresponding bar graph on the right panel represents the number of cells formed in colony formation, migration, or invasion assays. Data are presented as mean ± SD (*n* = 3). **F**–**J** HCT116 cells were transiently transfected with either Flag-SLC26A3 or a mock control plasmid. Western blotting and qPCR were performed to detect the expression levels of SLC26A3 (**F**, **G**). The transcript levels of SLC26A3 were normalized to the expression of GAPDH, and negative control cells were used as the baseline value of 1. The transfected cells were then evaluated for their proliferation ability using CCK-8 assay (**H**) and colony formation assay (**I**), as well as migration and invasion ability using Transwell assay (**J**). The corresponding bar graph on the right panel represents the number of cells formed in colony formation, migration, or invasion assays. Data are presented as mean ± SD (*n* = 3). **K**–**O** HCT116 cells were stably transfected with pLKO.1, SLC26A3-sh1, or SLC26A3-sh2 plasmids. Western blotting and qPCR were employed to detect the expression levels of SLC26A3 (**K**, **L**). The transcript levels of SLC26A3 were normalized to the expression of GAPDH, and negative control cells were used as the baseline value of 1. The transfected cells were then examined for their proliferation ability using CCK-8 assay (**M**) and colony formation assay (**N**), as well as their migration and invasion ability using Transwell assay (**O**). The corresponding bar graph on the right panel represents the number of cells formed in colony formation, migration, or invasion assays. Data are presented as mean ± SD (*n* = 3). **P**–**T** HCT116 cells were stably transfected with either SLC26A3-KO or a mock control plasmid. Western blotting and qPCR were performed to detect the expression levels of SLC26A3 **(P**, **Q**). The transcript levels of SLC26A3 were normalized to the expression of GAPDH, and negative control cells were used as the baseline value of 1. Proliferation ability was assessed using CCK-8 assay (**R**) and colony formation assay (**S**), while migration and invasion ability were evaluated using Transwell assay (**T**). The corresponding bar graph on the right panel represents the number of cells formed in colony formation, migration, or invasion assays. Data are presented as mean ± SD (*n* = 3). The data were analyzed using Student’s *t* test. Statistical significance was set at *p < 0.05, **p < 0.01, and ***p < 0.001.

As we observed a low expression of SLC26A3 in CRC tissues, our focus shifted towards exploring the potential effects of SLC26A3 on CRC cells. To this end, we designed lentiviral-encapsulated SLC26A3 overexpression plasmids and transfected them into HCT116 and Caco2 cell lines. Western blotting and qPCR assays were performed to confirm successful overexpression of SLC26A3 (Fig. 2A, B and S2A, B). We then evaluated the proliferation capability of the cells using CCK-8 and colony formation assays, which demonstrated that SLC26A3 overexpression inhibited the proliferation and colony formation ability of CRC cell lines (Fig. 2C, D and S2C, D). Furthermore, we conducted Transwell assays to assess the migration and invasion capabilities of these cells, revealing that SLC26A3 significantly suppressed the migration and invasion abilities of CRC cell lines (Fig. 2E and S2E). In addition, direct transfection with SLC26A3 plasmids into HCT116 cells also resulted in transient overexpression of SLC26A3 and subsequent inhibition of proliferation, migration, invasion, and colony formation abilities (Fig. 2F–J).

Additionally, we designed lentiviral-encapsulated SLC26A3 knockdown plasmids to be transfected into CRC cell lines. Crispr-Cas9-mediated SLC26A3 knockout was also performed on HCT116 cells to eliminate SLC26A3 expression. Conversely, downregulation or knockout of SLC26A3 significantly promoted proliferation, migration, invasion, and colony formation abilities (Fig. 2K–Q and S2F–J). Collectively, these findings indicate that SLC26A3 acts as a tumor suppressor in CRC and inhibits the malignant biological behaviors of CRC cells.

### SLC26A3 modulated the NF-κB signaling pathway in CRC

**Fig. 3:**
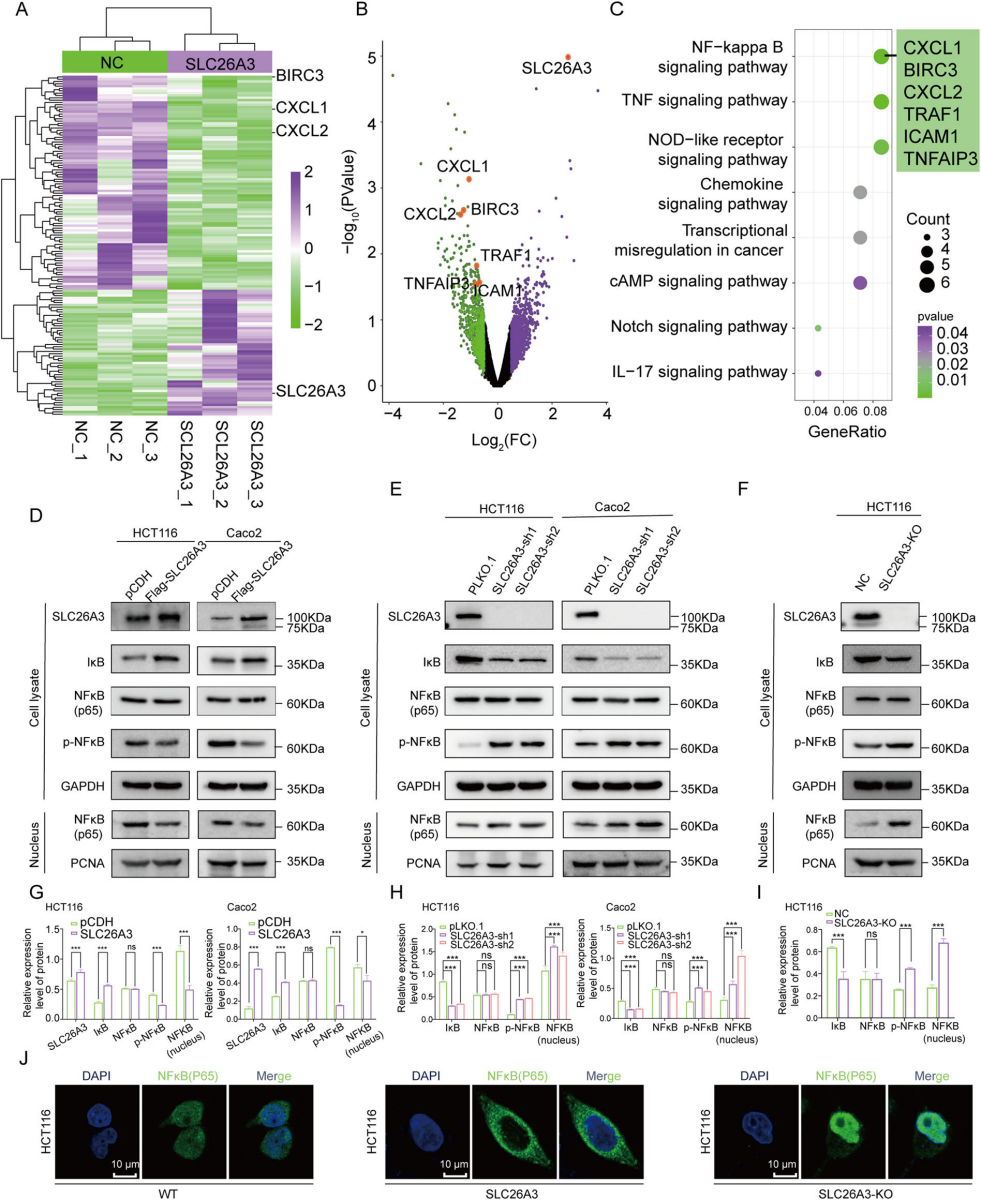
SLC26A3 modulated the NF-κB pathway. **A**, **B** Transcriptome sequencing was detected on SW480 cells stably transfected with SLC26A3 plasmid or mock control plasmid with three repetitions of each cell line. Significant differentially expressed genes on SLC26A3 overexpression in SW480 cells by RNA-seq are shown as a heatmap (**A**) and volcano (**B**). **C** KEGG Pathway enrichment analysis of the differentially expressed genes. **D** Western blotting analysis of NF-κB pathway, including NF-κB(p65), p- NF-κB and IκB protein levels in whole cell and nucleus of HC116 and Caco2 cells stably overexpressed SLC26A3. **E** Western blotting analysis of NF-κB pathway protein levels in whole cell and nucleus of HCT116 and Caco2 cells stably transfected with lentivirus-mediated SLC26A3-sh1, SLC26A3-sh2 plasmid or mock control plasmid. **F** Western blotting analysis of NF-κB pathway protein levels in whole cell and nucleus of SLC26A3-knockout HCT116 cells. **G**–**I** Quantitative analyses of western blotting results were shown in the graphs. GAPDH was used as a loading control for whole-cell lysates, and PCNA was used as a loading control for nuclear extracts. **J** Cellular localization of p65 in wild type HCT116 cells, SLC26A3 overexpression HCT116 cells and SLC26A3 knockout HCT116 cells. Data are presented as mean ± SD of three independent experiments. The data were analyzed using Student’s *t* test. Statistical significance was set at ns: not significant, *p < 0.05, **p < 0.01, and ***p < 0.001.

To elucidate the mechanism by which SLC26A3 inhibits malignant biological behaviors in CRC cells, we determined which biological pathways affected by SLC26A3 were associated with this function. Transcriptome sequencing was performed on SW480 cells stably transfected with SLC26A3 plasmid or mock control plasmids, with three repetitions for each cell line. Firstly, we analyzed the overall impact of SLC26A3 expression levels on whole gene expression levels and found 538 significantly downregulated genes (including CXCL1 and CXCL2, which are regulated by the NF-κB transcription factor). KEGG analysis then confirmed that genes differentially expressed in response to SLC26A3 downregulation were mainly associated with the NF-κB signaling pathway (Fig. 3A–C). Hence, we assessed the changes in key genes within the NF-κB signaling pathway subsequent to SLC26A3 regulation via qPCR and Western blotting assays. The results showed that CXCL1 and CXCL2 mRNA levels were significantly downregulated after SLC26A3 overexpression and upregulated after SLC26A3 knockout, while NF-κB (p65) and IκB mRNA levels remained unchanged (Fig. S3A, B). Furthermore, western blotting of whole cell protein revealed that p-NF-κB expression was significantly downregulated and IκB was markedly upregulated after SLC26A3 overexpression, with opposite results observed after SLC26A3 knockout. Interestingly, p65 expression did not change at the whole cell protein level. We also tested p65 expression levels in the nucleus and found that they inversely correlated with SLC26A3 expression levels (Fig. 3D–I and S3C, D). Additionally, we performed cellular immunofluorescence (IF) experiments to investigate the subcellular localization of p65 protein following SLC26A3 regulation. The results revealed an even distribution of p65 protein in the cytoplasm and nucleus of wild-type cell lines, whereas its localization shifted to the cytoplasm upon SLC26A3 overexpression and towards the nucleus upon SLC26A3 knockout (Fig. 3J), indicating a differential impact of SLC26A3 regulation on p65 subcellular localization. In summary, our data revealed that SLC26A3 inhibited NF-κB signaling pathway in CRC cells via influence in IκB protein expression and NF-κB/p65 nucleus translocation.

### SLC26A3 directly interacts with NHERF2 protein

**Fig. 4:**
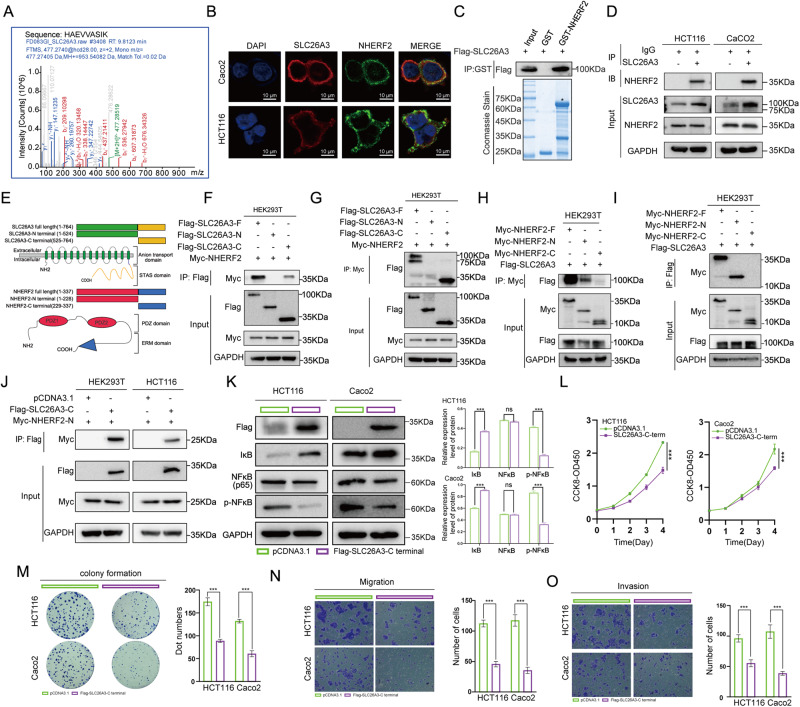
SLC26A3 directly interacted with NHERF2 protein. **A** NHERF2 was identified by mass spectrometry analysis from HEK293T cells overexpressing SLC26A3. **B** Cellular colocalization of SLC26A3 with NHERF2. In HCT116 cells and Caco2 cells co-transfected with Flag-SLC26A3 and Myc-NHERF2. The nucleus was stained by DAPI (blue), Flag-SLC26A3(red) and Myc-NHERF2(green) were observed by laser scanning confocal microscopy. Scale bar = 10 μM. **C** Reconstituted GST fusion NHERF2 protein directly bound with Flag-SLC26A3 observed in GST pulldown assay. **D** Co-IP analysis of the interaction between endogenous SLC26A3 and endogenous NHERF2 in HCT116 and Caco2 cells, by using SLC26A3 antibody (5 μg/mL, Santa Cruz Biotechnology, Inc) to IP SLC26A3. **E** Schematic illustration of SLC26A3 and NHERF2, showing the wild-type and truncated fragments of genes and their corresponding domains. **F**, **G** Co-IP analysis the binding of NHERF2 to SLC26A3 or SLC26A3 truncated fragments in HEK293T cells, co-transfected with plasmid coding for Flag-SLC26A3 or Flag-SLC26A3 truncated fragments together with NHERF2 plasmid, using Flag-tagged (**F**) and Myc-tagged (**G**) anti-beads respectively. **H**,**I** Co-IP analysis of the binding between SLC26A3 and NHERF2 or NHERF2 truncated fragments in HEK293T cells co-transfected with Myc-NHERF2 or Myc-NHERF2 truncated fragment plasmids together with SLC26A3 plasmid, using Myc-tagged (**H**) and Flag-tagged (**I**) anti-beads respectively. **J** Co-IP analysis of the binding between Flag-SLC26A3-C terminal fragment to Myc-NHERF2-N terminal fragment plasmid in HEK293T and HCT116 cells. **K**–**O** Function of the SLC26A3-C terminal was in CRC cells. Western blotting analysis of NF-κB pathway, including NF-κB, p-NF-κB, and IκB protein levels in HCT116 and Caco2 cells transiently transfected with Flag-SLC26A3-C terminal plasmid or mock control plasmid (**K**). GAPDH was used as a loading control. The malignant behaviors of transfected CRC cells, including proliferation(**L**), colony formation(**M**), migration (**N**) and invasion (**O**) were determined. Quantitative analysis was showed in the graphs. Data are presented as mean ± SD of three independent experiments. ns: not significant. *p < 0.05, **p < 0.01, ***p < 0.001 based on the Student’s *t* test.

To further explore the mechanisms underlying SLC26A3’s inhibition of the NF-κB signaling pathway, we performed mass spectrometry analysis coupled with Co-IP of SLC26A3 proteins to identify differentially expressed proteins in HEK293T cells transiently transfected with Flag-SLC26A3 plasmids. These analyses identified 308 proteins that might interact with SLC26A3 (Table S3), and we selected NHERF2 for further verification (Fig. 4A).

To investigate the potential interaction between SLC26A3 and NHERF2, immunofluorescence experiments were performed on HCT116 and Caco2 cells, which demonstrated that SLC26A3 colocalized mainly with NHERF2 at the cytomembrane (Fig. 4B). Then, the reconstituted GST fusion NHERF2 protein purified from prokaryotic expression system was directly interacted with SLC26A3 protein in vitro (Fig. 4C). Co-IP experiments were then conducted on HCT116, Caco2, and HEK293T cells, revealing that SLC26A3 interacts with both exogenous and endogenous NHERF2 proteins (Fig. S4A–C). Additionally, western blotting confirmed that endogenous SLC26A3 can interact with endogenous NHERF2 (Fig. 4D and S4D).

To investigate the molecular basis of the binding between SLC26A3 and NHERF2, several functional domain fragment plasmids of SLC26A3 and NHERF2 were constructed, as shown in the schematic diagram (Fig. 4E). The results of Co-IP showed that NHERF2 bound with wild type SLC26A3 and the C-terminal domain (STAS domain, 525–764 aa), but not with the N-terminal domain (Anion transport domain, 1–524 aa), suggesting that the interaction of SLC26A3 with NHERF2 is dependent on the C-terminal domain of SLC26A3 (Fig. 4F, G). Next, HEK293T cells were co-transfected with Flag-SLC26A3 plus Myc-NNHERF2 wild type or N-terminal of NHERF2 (PDZ domain, 1–228 aa) or C-terminal of NHERF2 (ERM domain, 229–324) for 48 h, and Co-IP was subsequently carried out. The results indicated that SLC26A3 could bind with wild type NHERF2 and the PDZ domain, but not with the ERM domain (Fig. 4H, I). Finally, HEK293T and HCT116 cells were co-transfected with C-terminal of SLC26A3 plus N-terminal of NHERF2 for 48 h, and Co-IP was subsequently carried out. The results indicated that the C-terminal of SLC26A3 could interact with the N-terminal of NHERF2 (Fig. 4J). These findings suggest that the C-terminal of SLC26A3 interacts with the N-terminal of NHERF2.

### Function of SLC26A3-C terminal in CRC cells

To further verify whether the SLC26A3-C terminus can also inhibit malignant biological behaviors in CRC, we investigated its influence on the NF-κB signaling pathway and malignant biological behaviors. Overexpression of the SLC26A3-C terminus notably downregulated p-NF-κB protein levels and upregulated IκB levels, fully revealing its inhibition of the NF-κB pathway (Fig. 4K). Proliferation (Fig. 4L), colony formation (Fig. 4M), migration (Fig. 4N), and invasion (Fig. 4O) of CRC cells were all significantly inhibited. In summary, our data demonstrate that the SLC26A3-C terminus inhibits malignant biological behaviors of CRC cells via downregulation of the NF-κB signaling pathway.

### NHERF2 protein deubiquinated the IκB protein

**Fig. 5:**
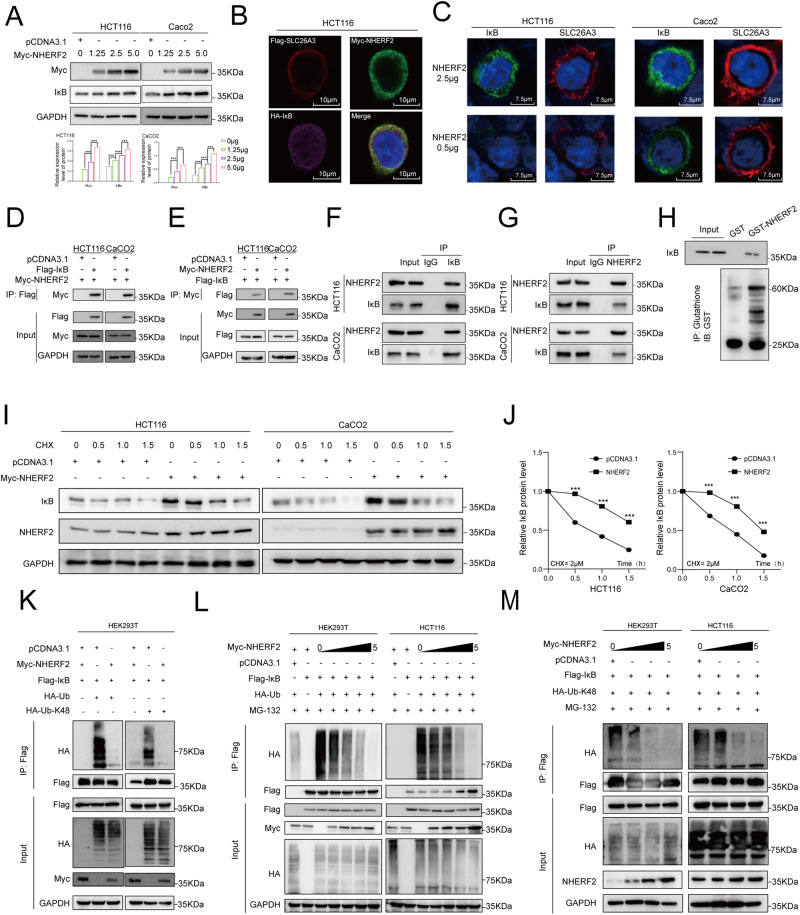
Effects of NHERF2 on the abundance of IκB protein and K48-linked polyubiquitination. **A** Western blotting analysis of IκB protein level in HCT116 cells and Caco2 cells transiently transfected with different amounts (0, 1.25, 2.5 and 5 μg) of Myc-NHERF2 plasmid. Quantitative analysis was showed in the graphs. **B** The location of three proteins in HCT116 cells. In HCT116 cells co-transfected with Flag-SLC26A3, HA- IκB and Myc-NHERF2. The nucleus was stained by DAPI (blue), Flag-SLC26A3(red), HA-IκB (pink) and Myc-NHERF2(green) were observed by laser scanning confocal microscopy. Scale bar = 10 μM. Cells incubated with antibodies against Alexa Flour 647-conjugaed Flag-Tag (1:200; Proteintech Group, Inc), Alexa Flour 594-conjugaed HA-Tag (1:200; Proteintech Group, Inc) and Alexa Flour 488-conjugaed Myc-Tag (1:400; Proteintech Group, Inc) overnight at 4 °C, next day DAPI (Abcam) was applied for nuclei staining, and cells were observed with a laser scanning confocal microscope TCS SP8 (Leica Microsystems, Wetzlar, Germany). **C** The expression level and location of SLC26A3 and IκB in CRC after transfected with different amount of Myc-NHERF2 plasmid. In HCT116 and Caco2 cells transfected with different amount of Myc-NHERF2 plasmid. The nucleus was stained by DAPI (blue), SLC26A3(red) and IκB (green) were observed by laser scanning confocal microscopy. Scale bar = 7.5 μM. Cells incubated with antibodies against SLC26A3(200 μg/mL; Santa Cruz Biotechnology, Inc) and IκB (1:400; Abcam) overnight at 4 °C. Next day, Alexa Flour 647-conjugaed goat anti-mouse secondary antibody (1:200; Abcam) and Alexa Flour 488-conjugaed goat anti-rabbit secondary antibody (1:200; Abcam) were used to incubate the cells. DAPI (Abcam) was applied for nuclei staining, and cells were observed with a laser scanning confocal microscope TCS SP8 (Leica Microsystems, Wetzlar, Germany). **D**, **E** Co-IP analysis of the interaction between exogenous NHERF2 and exogenous IκB in HCT116 cells and Caco2 cells transiently co-transfected with Flag-IκB and Myc-NHERF2 plasmid, and using Myc-tagged and Flag-tagged anti-beads respectively. **F**, **G** Co-IP analysis was performed to investigate the interaction between endogenous NHERF2 and endogenous IκB in HCT116 cells and Caco2 cells. **H** A GST pulldown assay demonstrated a direct binding between reconstituted GST-NHERF2 protein and Flag-IκB. **I** CRC cells were transfected with Myc-NHERF2 or mock control plasmid and were cultured for 36 h before being treated with CHX (40 μg/mL) for 0, 0.5, 1.0, 1.5 h. The IκB protein level was detected by Western blotting. **J** Quantitative analysis was showed in the graphs. **K** Co-IP analysis of the ubiquitination of IκB in HEK293T cells co-transfected with Flag-IκB plasmid, Myc-NHERF2 plasmid, HA-UB/HA-UB-K48 plasmid or mock control plasmid. **L**, **M** Co-IP analysis was also performed to investigate the ubiquitination of IκB in HEK293 and HCT116 cells that were co-transfected with varying amounts (0, 1.25, 2.5, and 5 μg) of Myc-NHERF2, and equal amounts of Flag-IκB and HA-UB or HA-UB-K48 plasmid for 48 h. Data are presented as mean ± SD (*n* = 3). ns: not significant. *p < 0.05, **p < 0.01, ***p < 0.001 based on the Student’s *t* test.

To investigate the potential effect of NHERF2 on IκB, we initially detected the protein level of IκB after transiently transfecting varying amounts of NHERF2 plasmids. The expression level of IκB was found to increase with increasing levels of NHERF2 gene expression in HCT116 and Caco2 cells (Fig. 5A). Notably, altering NHERF2 expression by overexpression or knockdown had no significant effect on the mRNA expression level of IκB (Fig. S5), suggesting that NHERF2-mediated upregulation of IκB protein occurs at the translational level rather than the transcriptional level of IκB.

To determine the subcellular localization of NHERF2 and IκB, we conducted IF experiments in HCT116 and Caco2 cells, which revealed that NHERF2 colocalized with IκB predominantly in the plasma membrane (Fig. S6). Moreover, IF experiments conducted in HCT116 cells to investigate the localization of NHERF2, IκB, and SLC26A3 demonstrated that these three proteins also primarily colocalized in the plasma membrane (Fig. 5B). Additionally, our IF results indicated that increasing NHERF2 expression coincided with an increase in SLC26A3 and IκB expression. Furthermore, SLC26A3 primarily increased in the cell membrane, while IκB mainly increased in the cytoplasm (Fig. 5C). Then, exogenous and endogenous co-immunoprecipitation (co-IP) assays were performed to identify potential interactions between NHERF2 and IκB. Our data indicated that NHERF2 indeed bound exogenous and endogenous IκB protein (Fig. 5D–G). Additionally, the reconstituted GST fusion NHERF2 protein purified from a prokaryotic expression system was found to directly interact with IκB protein in vitro (Fig. 5H).

To further investigate the effect of NHERF2 on IκB degradation, cycloheximide (CHX) was used to inhibit protein translation, and CRC cells transfected with Myc-NHERF2 plasmid or mock control plasmid were cultured with CHX. Our results showed that NHERF2 significantly inhibited the decrease of IκB protein level (Fig. 5I), ultimately prolonging the degradation time of IκB (Fig. 5J). IκB is a protein that undergoes Lys48(K48)-linked ubiquitination modification. To provide further evidence that IκB modification may be regulated by NHERF2, we co-transfected Flag-tagged IκB and Myc-tagged NHERF2 with two HA-tagged Ub (wild type Ub or K48-linkage-type-only Ub, HA-Ub or HA- K48) into HEK293T cells. Our results showed that both HA-Ub and HA-K48 were removed from IκB by NHERF2 (Fig. 5K), while the degree of deubiquitination of IκB was enhanced by increasing NHERF2 expression (Fig. 5L, M). Thus, our data indicated that NHERF2 can interact with and stabilize the IκB protein by removing K48 polyubiquitination chains from IκB.

### NHERF2 inhibited the malignant behaviors of CRC cells

**Fig. 6:**
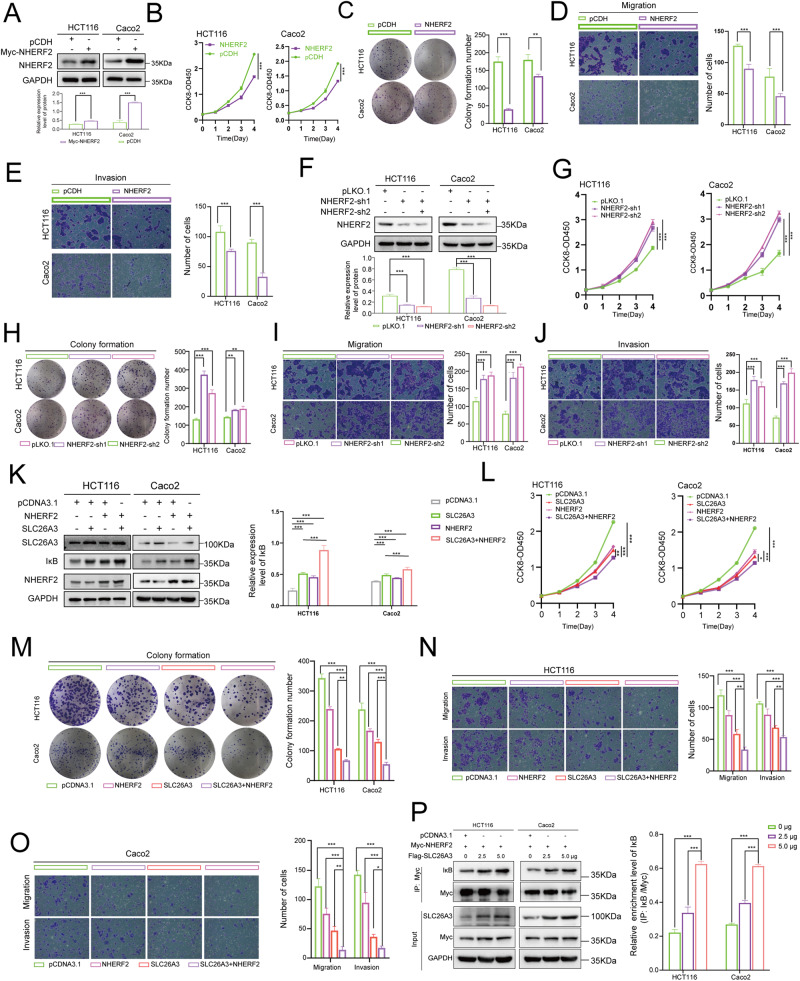
Effects of NHERF2 on the malignant behaviors of CRC cells combined with IκB enhanced by SLC26A3. **A**–**E** HCT116 and Caco2 cells were stably transfected with either NHERF2 or a mock control plasmid. Western blotting was performed to detect the expression levels of SLC26A3 (**A**). Quantitative analyses of western blotting results were shown in the graphs. And GAPDH was used as a loading control. The transfected cells were further assessed for their proliferation ability using CCK-8 assay (**B**) and colony formation assay (**C**), as well as migration and invasion ability using Transwell assay (**D**, **E**). The corresponding bar graph on the right panel represents the number of cells formed in colony formation, migration, or invasion assays. Data are presented as mean ± SD (*n* = 3). **F**–**J** HCT116 and Caco2 cells were stably transfected with pLKO.1, NHERF2-sh1, or NHERF2-sh2 plasmids. Western blotting was employed to detect the expression levels of SLC26A3 (**F**). Quantitative analyses of western blotting results were shown in the graphs. And GAPDH was used as a loading control. The transfected cells were then examined for their proliferation ability using CCK-8 assay (**G**) and colony formation assay (**H**), as well as their migration and invasion ability using Transwell assay (**I, J**). The corresponding bar graph on the right panel represents the number of cells formed in colony formation, migration, or invasion assays. Data are presented as mean ± SD (*n* = 3). **K**–**O** HCT116 and Caco2 cells were transiently transfected with Flag-SLC26A3 and/or Myc-NHERF2 or mock control plasmid. Western blotting was performed to detect the expression levels of IκB (**K**). Quantitative analyses of western blotting results were shown in the graphs. And GAPDH was used as a loading control. Malignant behaviors of transfected CRC cells, including proliferation (**L**), colony formation (**M**), migration (**N**) and invasion (**O**) were measured. The corresponding bar graph on the right panel represents the number of cells formed in colony formation, migration, or invasion assays. Data are presented as mean ± SD (*n* = 3). **P** Co-IP analysis was performed to investigate the IκB protein level in HCT116 and Caco2 cells that were transiently co-transfected with Myc-NHERF2 plasmid and varying amounts (0, 2.5, 5 μg) of Flag-SLC26A3 plasmid. Quantitative analyses of western blotting results were shown in the right panel. And Myc-tagged was used as a loading control. Data are presented as mean ± SD (*n* = 3). ns: not significant. *p < 0.05, **p < 0.01, ***p < 0.001 based on the Student’s *t* test.

Given the controversial nature of NHERF2’s function, we sought to ascertain its potential impact on CRC cells. To this end, we devised a lentiviral vector-based approach for inducing overexpression of NHERF2 in HCT116 and Caco2 CRC cell lines. Western blotting analyses were employed to verify successful NHERF2 overexpression (Fig. 6A). Subsequent evaluation showed that NHERF2 overexpression exerted inhibitory effects on CRC cell proliferation (Fig. 6B) and significantly curbed tumor formation as indicated by colony formation assays (Fig. 6C). Moreover, Transwell assays revealed that NHERF2 overexpression effectively abrogated migration and invasion of CRC cells relative to controls (Fig. 6D, E).

Moreover, we devised lentiviral vectors that encapsulated NHERF2 knockdown plasmids for transfection into CRC cell lines. Intriguingly, in NHERF2-knockdown CRC cells (Fig. 6F), we observed a marked increase in their proliferation (Fig. 6G), colony formation ability (Fig. 6H), migration and invasion capacity (Fig. 6I, J). Collectively, our findings provide compelling evidence that NHERF2 plays a vital role in CRC as a tumor suppressor by suppressing the malignant biological behaviors of CRC cells.

### SLC26A3 enhanced NHERF2 and IκB interaction

Based on the interaction between SLC26A3 and NHERF2, we formulated the hypothesis that SLC26A3 may promote the association of NHERF2 with IκB. To validate this conjecture, we initially explored whether SLC26A3 modulated the binding of NHERF2 to IκB. Our data demonstrate that SLC26A3 enhances the expression of IκB in the presence of NHERF2 (Fig. 6K), thereby impeding the malignant behaviors of CRC cells, including proliferation (Fig. 6L), colony formation (Fig. 6M), migration, and invasion (Fig. 6N, O). In co-transfection experiments with varying amounts of SLC26A3 plasmid and Myc-NHERF2 plasmid, we observed an increase in the level of IκB protein immunoprecipitated by the Myc-tagged antibody (Fig. 6P). Taken together, our findings suggest that SLC26A3 collaborates with NHERF2 to promote IκB degradation.

### SLC26A3 is a direct transcriptional target of NF-κB/p65

**Fig. 7:**
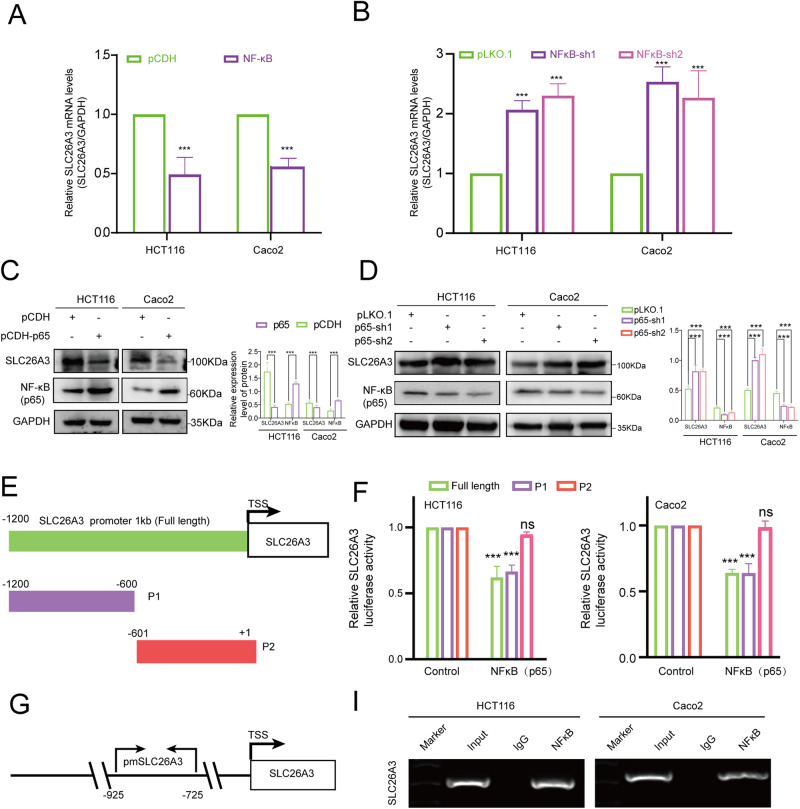
Ectopic expression of NF-kB subunit p65 inhibited SLC26A3 expression. **A**, **B** The relative mRNA expression level of SLC26A3 after p65 overexpression or knockdown in HCT116 and Caco2 cells. The transcript levels of SLC26A3 were normalized to the expression of GAPDH, and negative control cells were used as the baseline value of 1. Data are presented as mean ± SD (*n* = 3). **C**, **D** The protein expression level of SLC26A3 after p65 overexpression or knockdown in HCT116 and Caco2 cells. Quantitative analyses of western blotting results were shown in the graphs. And GAPDH was used as a loading control. Data are presented as mean ± SD (*n* = 3). **E** A diagram showed the relative positions of full length and fragment in SLC26A3 promoter. **F** The luciferase activity of the SLC26A3 promoter was analyzed. Data are presented as mean ± SD (*n* = 3). **G** A diagram showed the PCR tested the relative positions of SLC26A3 position. **I** The agarose gel showed the Chip-PCR results. Quantitative analysis was showed in the graphs. Data are presented as mean ± SD of three independent experiments. ns: not significant. *p < 0.05, **p < 0.01, ***p < 0.001 based on the Student’s *t* test.

As we mentioned above, SLC26A3 increases the protein expression level of IκB by removing its ubiquitination through NHERF2 and inhibits the dissociation of p65 from the IκB/p65/p50 complex, thereby reducing the nuclear translocation of p65. Furthermore, public databases predicted that the promoter of SLC26A3 may interact with p65, prompting us to investigate whether NF-κB/p65 regulates the expression of SLC26A3 and directly binds to the SLC26A3 gene promoter element. To this end, we designed lentiviral vectors for p65 overexpression and knockdown and transfected them into CRC cell lines HCT116 and Caco2, respectively. Western blotting and quantitative PCR assays confirmed that p65 had a satisfactory effect on SLC26A3 expression. Intriguingly, p65 overexpression significantly decreased both SLC26A3 protein and mRNA levels, whereas p65 knockdown produced opposite results (Fig. 7A–D). Given that p65 modulates SLC26A3 expression, we next examined the direct interaction between p65 and the SLC26A3 promoter region. We cloned the full-length (FL; 1.2 kb) and two fragments (P1 and P2) of the SLC26A3 promoter (Fig. 7E) into a luciferase reporter plasmid. These three types of luciferase reporter plasmids were then co-transfected with empty plasmids (Control), p65 plasmids, and pRL-TK into Caco2 and HCT116 cells, respectively. The luciferase reporter data showed that FL and P1 fragments directly bound to p65 (Fig. 7F). To confirm the interaction between p65 and the P1 fragment, ChIP-PCR assays were conducted to evaluate the genomic occupancy of p65 at the P1 sequence. Our RT-PCR analysis of the chromatins pulled down by p65 antibodies and total DNA revealed a preferential enrichment of p65 occupancy at the P1 sequence (Fig. 7I), strongly suggesting that p65 directly regulates SLC26A3 transcription.

### SLC26A3 suppressed tumorigenesis in vivo

**Fig. 8:**
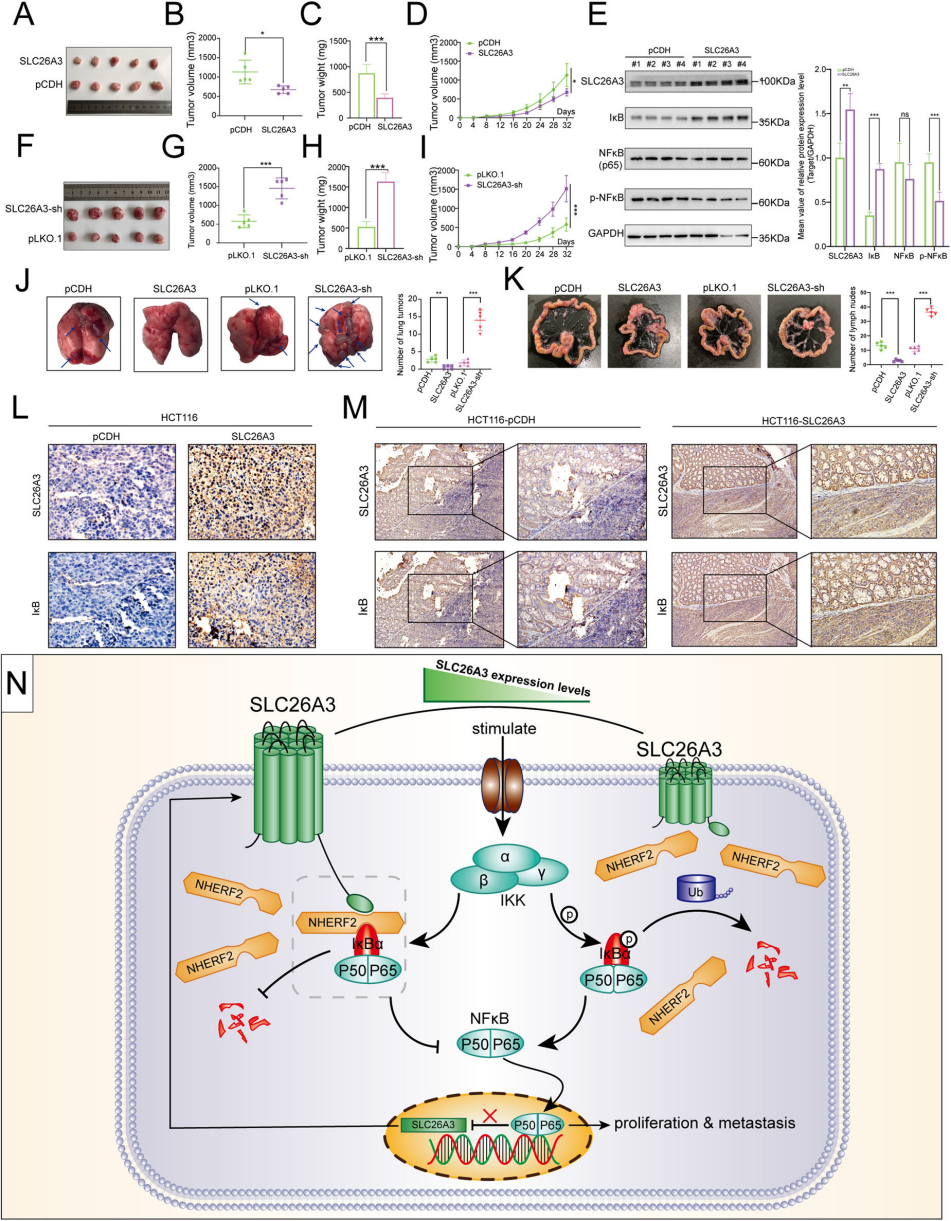
Tumor suppressor role of SLC26A3 analyzed in tumor xenografts models. **A**–**D** Subcutaneous injection of HCT116 cells stably transfected SLC26A3 plasmid or mock control plasmid. The image of tumors (**A**), tumor volume (**B**), tumor weight (**C**), and tumor growth curves (**D**). 5 mice for each experimental group. **E** Western blotting analysis IκB, NF-κB(p65) and p- NF-κB protein levels measured in extracted protein from each tumor tissue. **F**–**I** Subcutaneous injection of HCT116 cells stably transfected sh-SLC26A3 or mock control plasmid. The image of tumors (**F**), tumor volume (**G**), tumor weight (**H**), and tumor growth curves (**I**). 5 mice for each experimental group. **J** Lung excised from mice that performed tail vein injection of HCT116 cells stably transfected SLC26A3 plasmid, sh-SLC26A3 or mock control plasmid. 5 mice for each experimental group**. K** mesentery excised from mice after orthotopic tumor transplantation of four kinds of subcutaneous tumors. 5 mice for each experimental group**. L** Representative IHC images of SLC26A3 and IκB protein staining in Subcutaneous tumor tissues. **M** Representative IHC images of SLC26A3 and IκB protein staining in tumor tissues of orthotopic tumor. **N** Schematic representation of the major molecular mechanisms. The data are shown as the mean ± SD. *p < 0.05, **p < 0.01, ***p < 0.001 based on the Student’s *t* test.

To investigate the effect of the SLC26A3 in animal model, xenograft models were established by injecting stable overexpression SLC26A3 HCT116 cells, knockdown SLC26A3 HCT116 cells and their respective vector‐transfected HCT116 cells into subcutaneous tissues of nude mice. The solid tumors excised from each group were accessed, and the results showed that solid tumors stable overexpression with SLC26A3 were smaller than corresponding control group (Fig. 8A). Additionally, further research of the excised solid tumor indicated that the overexpression of SLC26A3 significantly decreased the weight and volume of solid tumors (Fig. 8B, C). Growth curve of the subcutaneously formed tumors revealed that overexpression of SLC26A3 significantly inhibited the growth of xenograft tumors compared to control tumors (Fig. 8D). SLC26A3 over-expression group was verified by detecting the extracted tumors to verify the expression levels of SLC26A3 via western blotting. Meanwhile, compared to control group tumors, the expression level of NF-κB remained unchanged, while the expression level of p-NF-κB was significantly decreased and that of IκB was significantly increased (Fig. 8E). On the contrary, the solid tumors excised from each group were accessed, and the results showed that solid tumors stable knockdown with SLC26A3 were bigger than corresponding control group (Fig. 8F). In additional, further research on the excised solid tumor revealed that knockdown of SLC26A3 significantly increased both the weight and volume of solid tumors. (Fig. 8G, H). Growth curve of the subcutaneously formed tumors showed that knockdown of SLC26A3 significantly promoted the growth of xenograft tumors compared to control tumors (Fig. 8I).

To further confirm the effect of SLC26A3 on the processes of metastasis in vivo, HCT116 cells with SLC26A3 overexpression or knockdown and their corresponding control cells were injected into the nude mice via tail vein to follow the metastasis. After 6 weeks, the lungs were excised from each group of mice. Our data showed that, mice injected with SLC26A3 overexpression cells reduced lung metastasis, instead, mice injected with SLC26A3 knockdown cells enhanced lung metastasis (Fig. 8J).

Metastatic model simulating human colorectal cancer was constructed by the orthotopic transplantation tumor of four kinds of subcutaneous tumor into colon wall of nude mice. Six weeks after transplant, mesentery was excised and the number of swollen lymph nodes formed on the mesentery surfaces were counted. Our data revealed that mice transplanted SLC26A3 overexpression solid tumor reduced lymph node metastasis, conversely, SLC26A3 knockdown solid tumor enhanced lung metastasis (Fig. 8K).

For the subcutaneous tumor, the IHC revealed that the expression level of IκB was positively correlated with SLC26A3 protein levels (Fig. 8L and Fig. S7A). For the orthotopic transplantation tumor, IHC analysis showed that in solid tumors with stable overexpression of SLC26A3, the expression level of IκB was higher compared to the control group. However, in the normal colon mucosa of mice, the expression levels of IκB were similar between the two groups. These results indicate a positive correlation between IκB and SLC26A3 protein levels (Fig. 8M and Fig. S7B, C).

In summary, NHERF2 was identified as a novel interacting protein of SLC26A3, and it also combined with IκB protein and maintained its stability via removing ubiquitination modification. Mechanistically, SLC26A3 enhanced the interaction between NHERF2 and IκB reducing its degradation. And this process inhibited p65 dissociation from IκB/p65/p50 complex and decreased the translocation of p65 from the cytoplasm to nucleus. Further, NF-κB/p65 bound directly to the promoter of SLC26A3 and reduced its mRNA expression. Thus, SLC26A3 inhibited the nucleus translocation of NF-κB/p65 promoting the transcription of SLC26A3, thereby establishing a positive regulatory feedback loop to suppresses the malignant biology of CRC cells. (Fig. 8N).

## Discussion

SLC26A3, a gene that encodes a protein known to be predominantly expressed in the colorectum and specifically located in the lumen brush border membrane of colorectal epithelial tissue [26] Previous studies have indicated that SLC26A3 is downregulated in CRC tissues, and the expression level of SLC26A3 is inversely correlated with the progression of colorectal cancer according to clinicopathological stage [27]. Additionally, SLC26A3, particularly its STAS domain, has been demonstrated to inhibit proliferation in colorectal cancer cells, indicating a growth regulatory role [28]. However, no studies have explored the impact of SLC26A3 on CRC cell invasion and migration. In this study, we observed that SLC26A3 expression was significantly lower in CRC tissues compared to normal colorectal tissues. Moreover, high levels of SLC26A3 expression were positively correlated with improved prognosis in CRC patients. Our findings also revealed that SLC26A3 expression levels were associated with clinical characteristics in CRC tissues. Furthermore, we demonstrated that SLC26A3 overexpression inhibited CRC cell proliferation, invasion, migration, and colony formation, while SLC26A3 knockdown or knockout had the opposite effect. Transient transfection of Flag-SLC26A3-STAS domain plasmid resulted in reduced malignant biological behaviors in colorectal cancer cells. Collectively, our results suggest that SLC26A3 acts as a tumor suppressor in CRC and downregulation of its expression may serve as a molecular biomarker for identifying CRC patients at high risk for poor prognosis.

Tumors often exhibit a significant reduction in interstitial pH compared to healthy tissue, reaching a low level [29]. Tumor acidity is a crucial driver of cancer progression as it contributes to the selection of malignant cancer cells and affects the composition and function of stromal cells in the tumor microenvironment [30]. Research indicates that acidosis weakens the anti-tumor effect and promotes immune evasion [31], whereas bicarbonate transporters can improve the acidic tumor environment and suppress tumor proliferation. Wen et al. reported that downregulation of bicarbonate transporter expression enhances the ability of renal cell carcinoma to proliferate and metastasize [32]. Similarly, Lee et al. demonstrated that the bicarbonate-transporting SLC26A6 can inhibit cellular migration [33]. SLC26A3, a bicarbonate transporter mainly expressed in the digestive system, inhibits the proliferation and metastasis of CRC cells in our study, and we also confirm that its C-terminal domain can suppress CRC progression. However, whether its anion transport domain plays a critical role in tumor suppression has not been investigated separately. As a bicarbonate transporter, SLC26A3 may also have effects on tumor suppression via its ion transport domain. Nevertheless, there is limited research in this area, hence further studies are warranted to elucidate its precise mechanism of action.

Despite its clinical relevance, the molecular mechanism of SLC26A3 in regulating the malignant biological behaviors of colorectal cancer cells remains largely unknown. However, Di Stadio et al. reported that SLC26A3 acts on the MAP kinases pathway to maintain mucosal integrity and prevent gastric cancer [34]. Furthermore, the expression of the SLC26A3 gene may be influenced by the PI3K/AKT pathway in the mammalian intestine [35, 36]. Hence, to elucidate the molecular mechanism of SLC26A3 in CRC progression, we overexpressed the SLC26A3 gene in colorectal cancer cell lines and performed RNA sequencing on cells with SLC26A3 overexpression. Interestingly, we found that the NF-κB signaling pathway was downregulated in CRC cells expressing the SLC26A3 gene. We also observed that overexpression and deletion of the SLC26A3 gene in CRC cell lines inactivated and activated the NF-κB signaling pathway, respectively. Additionally, SLC26A3 contains a C-terminal PDZ-binding motif [37], which plays a crucial role in assembling regulators by anchoring to PDZ scaffold proteins [38, 39]. Our study results suggest that SLC26A3 directly interacts with NHERF2 via the PDZ domain and that this interaction inhibits the biological behavior of CRC cancer cells by inactivating the NF-κB signaling pathway.

Belonging to the NHERF family, NHERF2 contains two PDZ domains that act as protein–protein interacting sequences [17–19], and scaffold proteins that modulate cell functions by coordinating regulators and second messengers [40]. Multiple studies have highlighted the crucial role of NHERF2 in cells. To explore its function in colorectal cancer cells, we conducted experiments, which showed that NHERF2 can inhibit the malignant behaviors of colorectal cancer cells. Bhattacharya et al. also observed that NHERF2 negatively regulates the expression of c-myc and cyclin D1 to inhibit cell proliferation [23]. And NHERF2 has also been shown that it can suppress tumor via recruit PTEN to inhibit the activation of the PI3K [22]. However, inconsistent results were reported in a study by Chris Yun et al., who found that NHERF2 positively regulates cancer cell proliferation in CRC cells [21]. We believe that the discrepancies in experimental results are due to differences in research models and methods. In this study, we overexpressed and knocked down NHERF2 in two CRC cell lines and found that NHERF2 inhibits the proliferation, migration, and invasion of CRC cells. Consistent results were obtained in both cell lines, indicating the reliability of our findings. Based on our results, we conclude that NHERF2 can inhibit the proliferation, migration, and metastasis of CRC cells, thereby providing new insights into the gene’s role in colorectal cancer.

Moreover, our study has demonstrated a direct interaction between NHERF2 and IκB, with significant implications for the regulation of the NF-κB signaling pathway in CRC. This protein-protein interaction suggests a potential mechanism for regulating proliferation and metastasis, which are critical processes in CRC progression. By identifying this interaction, our study highlights the importance of comprehending the complex network of interactions involved in the NF-κB signaling pathway to develop targeted therapeutic interventions for CRC. These findings could potentially provide new evidence for developing novel therapies that target the NHERF2-IκB interaction to modulate the NF-κB-mediated response, ultimately improving the prognosis of CRC patients. Previous studies have reported that the PDZ domain can alter the stability and expression of proteins that interact with it [41–43]. In our study, we showed that increased IκB expression resulting from NHERF2 overexpression depends on IκB deubiquitination. We showed that SLC26A3 promoted NHERF2 interaction with IκB in colorectal cancer cells. Furthermore, we found that the mRNA expression of IκB was not altered by NHERF2, indicating that its expression can be regulated at the post-translational level by NHERF2, but not transcriptional regulation. Our study also revealed that NHERF2 interacts with IκB to prevent IκB degradation and stabilize its expression by removing ubiquitination.

IκB plays a crucial role in the antitumor function of NHERF2. Our study data revealed that NHERF2 decreases IκB ubiquitination, which is associated with the inhibition of malignant behavior of tumor cells, such as cell proliferation, necrosis, and metastasis [44, 45]. In this study, we determined that NHERF2 interacts with IκB and decreases the level of IκB ubiquitination. Although IκB directly binds to NF-κB and masks its nuclear sites to regulate the translocation of NF-κB, phosphorylation, ubiquitination, and degradation of IκB release p65 from the IκB/p65/p50 protein complexes, which then translocates to the nucleus [46] to regulate multiple downstream genes.

Furthermore, we demonstrated the presence of a feedback loop between SLC26A3 and NF-κB/p65, which contributes to inhibiting the malignant behaviors of CRC cells. Additionally, we found a negative correlation between the expression levels of SLC26A3 and NF-κB/p65. We investigated the possible mechanisms by which NF-κB/p65 decreases SLC26A3 and performed dual-luciferase reporter system and Chip assays to assess the effect of the predicted NF-κB/p65 binding site in the SLC26A3 promoter region. Our data showed that NF-κB/p65 directly binds to the SLC26A3 promoter at the -925 to -725bp region. Ding and Kumar [9, 47], also reported a similar finding, indicating that NF-κB decreases SLC26A3 expression through its nucleus translocation.

In conclusion, our study has unraveled a previously unrecognized molecular mechanism that explains how membrane proteins inhibit CRC malignant biological behaviors and act as suppressive factors of CRC. We hypothesize that SLC26A3 plays a critical role in tumor inhibition via the SLC26A3/NHERF2-IκB/NF-κB/p65-SLC26A3 feedback loop. SLC26A3 promotes NHERF2 binding to IκB, which reduces IκB degradation by decreasing its ubiquitination levels. This, in turn, leads to the inhibition of downstream NF-κB/p65 dissociation from the IκB/p65/p50 complex and subsequent translocation to the nucleus, thus relieving transcriptional inhibition of NF-κB/p65 on SLC26A3. Our findings offer new insighs into the transcriptional and regulatory relationships between SLC26A3 and IκB, highlighting a potential therapeutic target for blocking CRC progression.

## Availability of data and materials

The RNA-seq data (GSE227981) underlying this article are currently subject to an embargo, which will last for a period of 3 years until Mar 21, 2027. After the embargo period expires, the data will be made available in a repository. You can access the data and find additional information through the following embargo link: https://www.ncbi.nlm.nih.gov/geo/query/acc.cgi.

### Supplementary information

Table S3

SUPPLEMENTARY MATERIAL
