# Supplementary material for: SLC26A3/NHERF2-IκB/NFκB/p65 feedback loop suppresses tumorigenesis and metastasis in colorectal cancer
Source: Oncogenesis. 2023 Aug 12;12(1):41. doi: 10.1038/s41389-023-00488-w (PMC10423209; doi:10.1038/s41389-023-00488-w)
Supplement: Supplementary file 2 — SUPPLEMENTARY MATERIAL [file 41389_2023_488_MOESM2_ESM.docx]

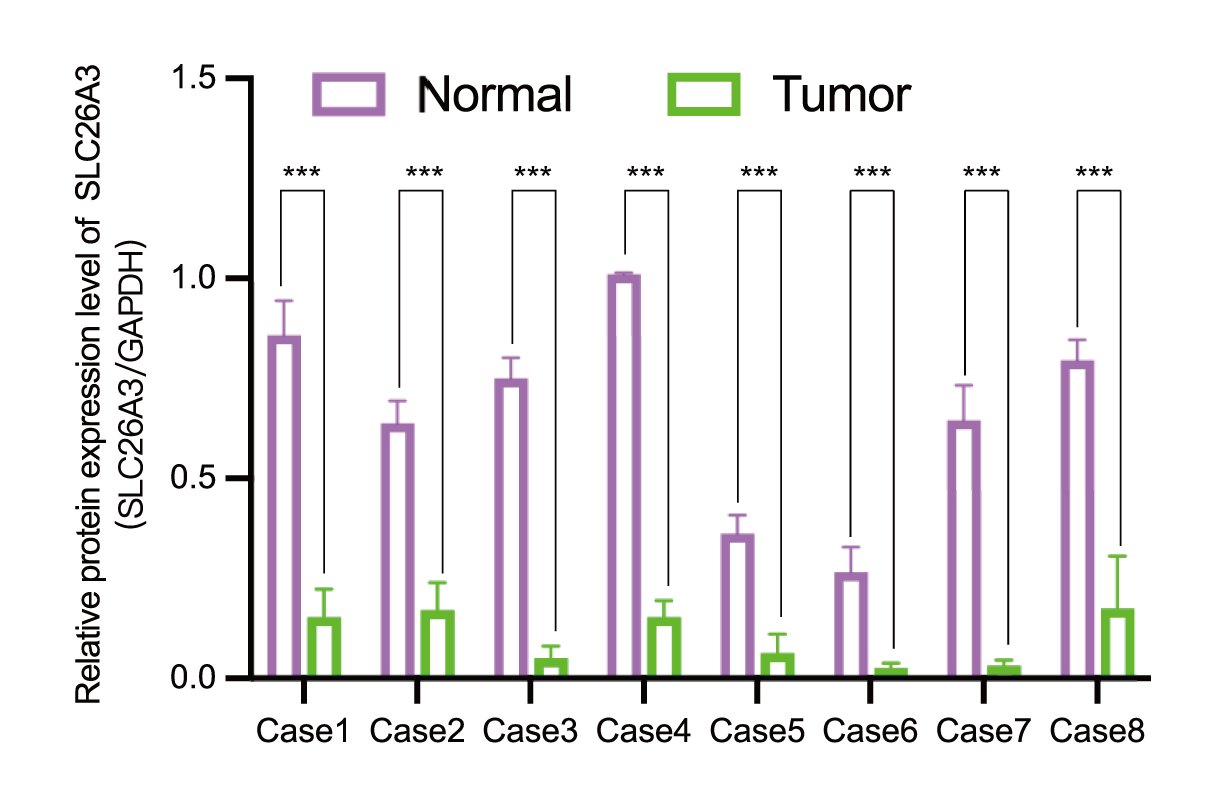


Fig.S1 The relative protein expression level of SLC26A3 in CRC tissues. GAPDH was used as a loading control. Data are presented as mean ± SD of three independent experiments. The data were analyzed using Student’s t-test. Statistical significance was set at ns: not significant, *p < 0.05, **p < 0.01, and ***p < 0.001.


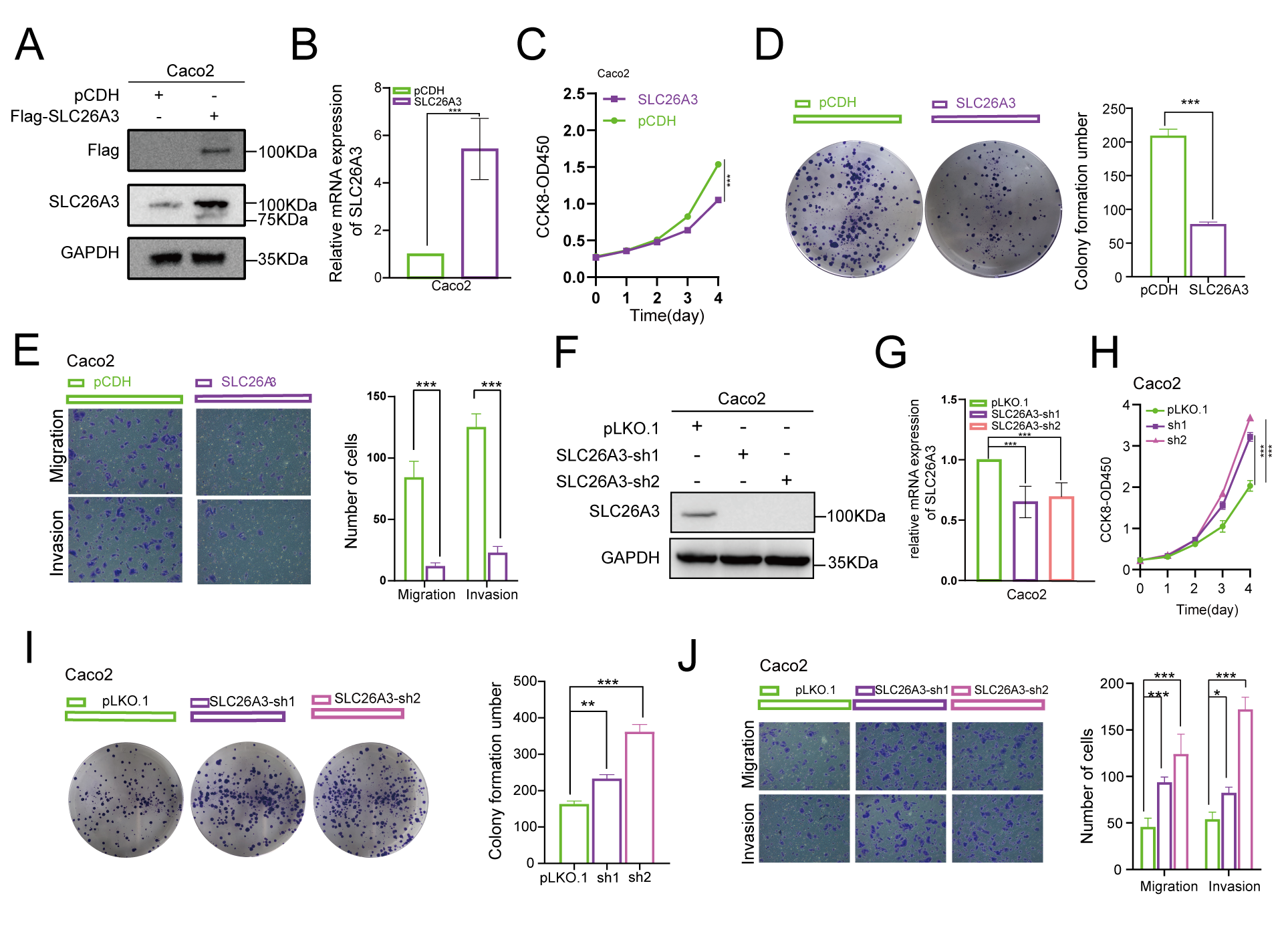


Fig.S2 SLC26A3 inhibited the malignant behaviors of CRC cells. (A-E) CaCO2 cells were stably transfected with either Flag-SLC26A3 or a mock control plasmid. Western blotting and qPCR were performed to detect the expression levels of SLC26A3 (A, B). The transcript levels of SLC26A3 were normalized to the expression of GAPDH, and negative control cells were used as the baseline value of 1. The transfected cells were further assessed for their proliferation ability using CCK-8 assay (C) and colony formation assay (D), as well as migration and invasion ability using Transwell assay (E). The corresponding bar graph on the right panel represents the number of cells formed in colony formation, migration, or invasion assays. Data are presented as mean ± SD (n = 3). (F-J) CaCO2 cells were stably transfected with pLKO.1, SLC26A3-sh1, or SLC26A3-sh2 plasmids. Western blotting and qPCR were performed to detect the expression levels of SLC26A3 (F, G). The transcript levels of SLC26A3 were normalized to the expression of GAPDH, and negative control cells were used as the baseline value of 1. The transfected cells were then evaluated for their proliferation ability using CCK-8 assay (H) and colony formation assay (I), as well as migration and invasion ability using Transwell assay (J). The corresponding bar graph on the right panel represents the number of cells formed in colony formation, migration, or invasion assays. Data are presented as mean ± SD (n = 3). The data were analyzed using Student’s t-test. Statistical significance was set at *p < 0.05, **p < 0.01, and ***p < 0.001.


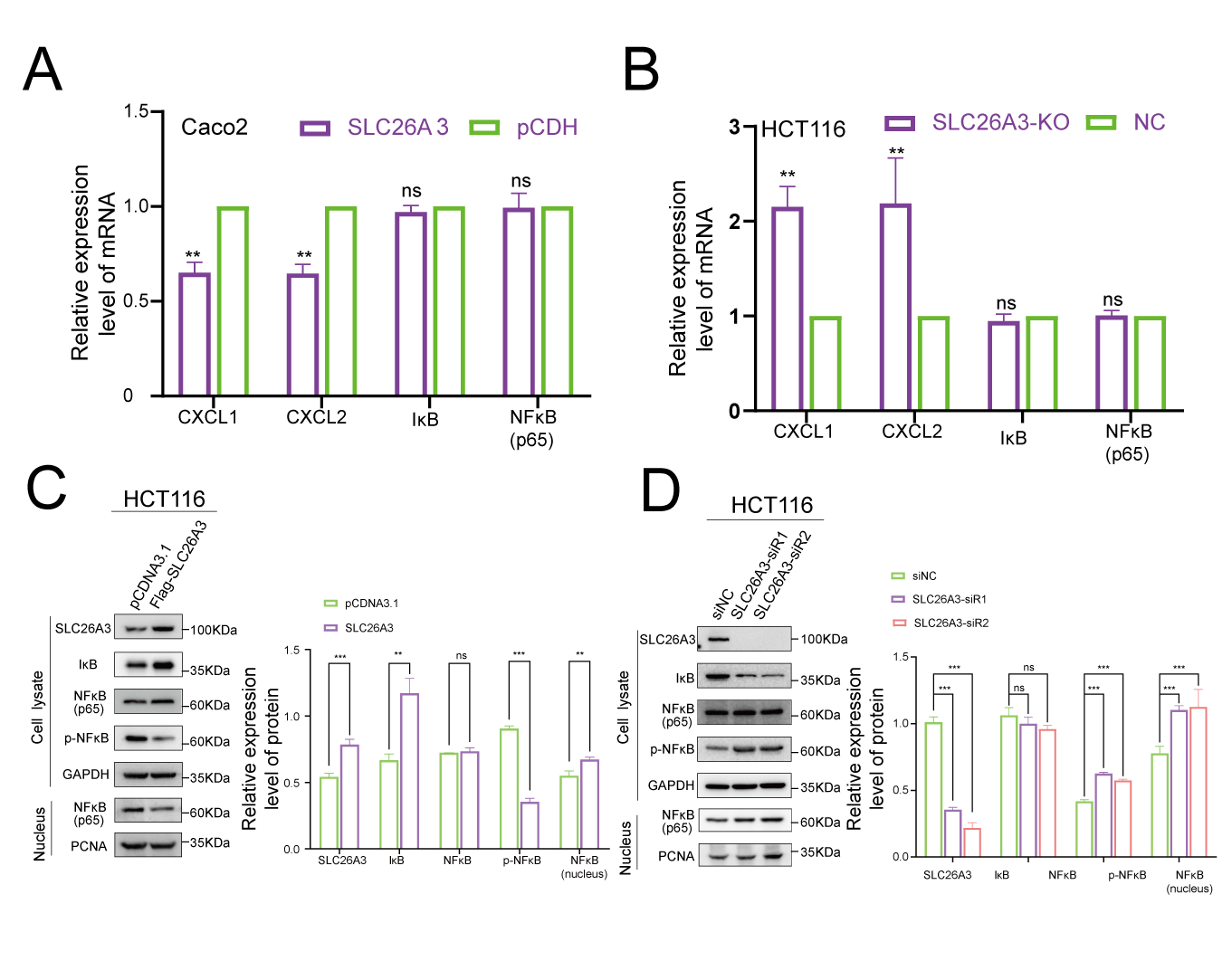


Fig.S3 SLC26A3 modulated the NF-κB pathway. (**A, B)** The relative mRNA expression level of NF-κB/p65, IκB and p65 downstream target genes CXCL1 and CXCL2 on SLC26A3 overexpression or knockout in CRC cells. Data are presented as mean ± SD (n = 3). (**C, D)** Western blotting analysis of NF-κB pathway, including NF-κB(p65), p-NF-κB and IκB protein levels in whole cell and nucleus of HCT116 transiently overexpression (C) or and knockdown SLC26A3 (D) Quantitative analyses of western blotting results were shown in the graphs. GAPDH was used as a loading control for whole-cell lysates, and PCNA was used as a loading control for nuclear extracts. Data are presented as mean ± SD of three independent experiments. The data were analyzed using Student’s t-test. Statistical significance was set at ns: not significant, *p < 0.05, **p < 0.01, and ***p < 0.001.


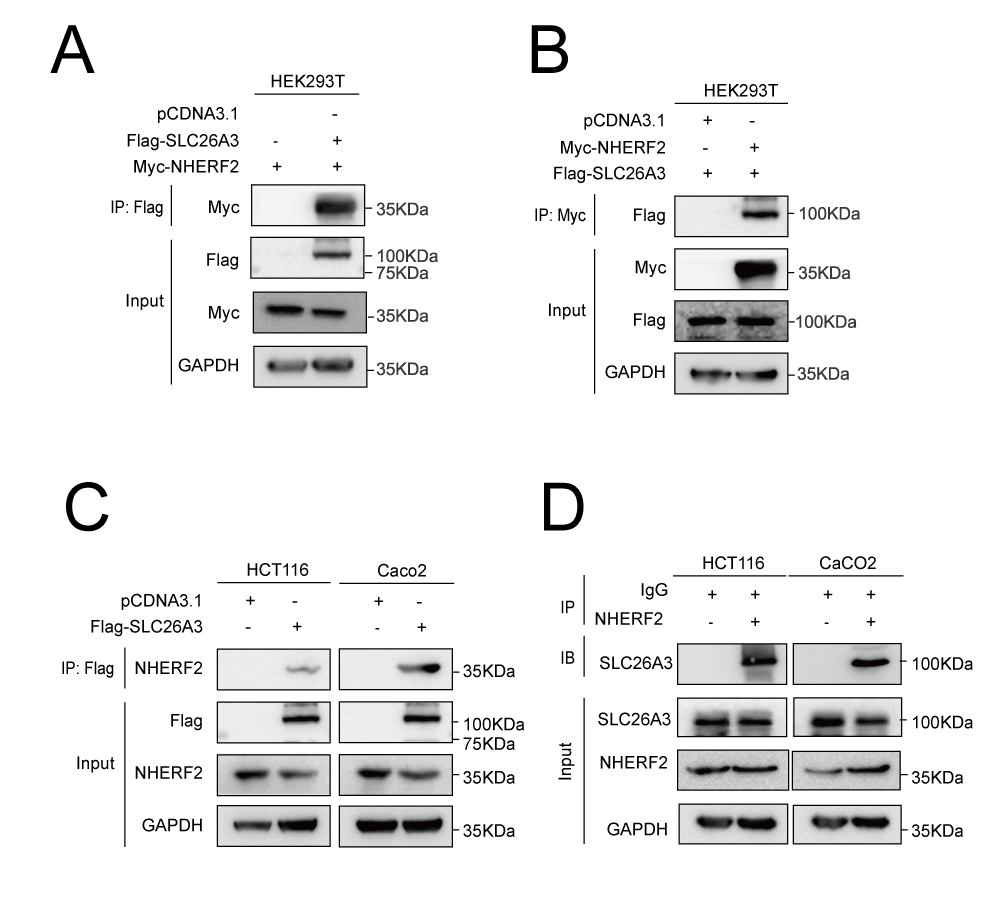


Fig.S4 NHERF2 interacted with SLC26A3 protein. (A, B) Co-IP analysis of the interaction between exogenous SLC26A3 and exogenous NHERF2 in HEK293T cells transiently co-transfected with Flag-SLC26A3 and Myc-NHERF2 plasmid. (C) Co-IP analysis of the interaction between exogenous SLC26A3 and endogenous NHERF2 in Caco2 and HCT116 cells transiently transfected with Flag-SLC26A3 or mock control plasmid. (D) Co-IP analysis of the interaction between endogenous SLC26A3 and endogenous NHERF2 in HCT116 and Caco2 cells by using NHERF2 antibody (5μg/mL, Santa Cruz Biotechnology, Inc) to IP NHERF2


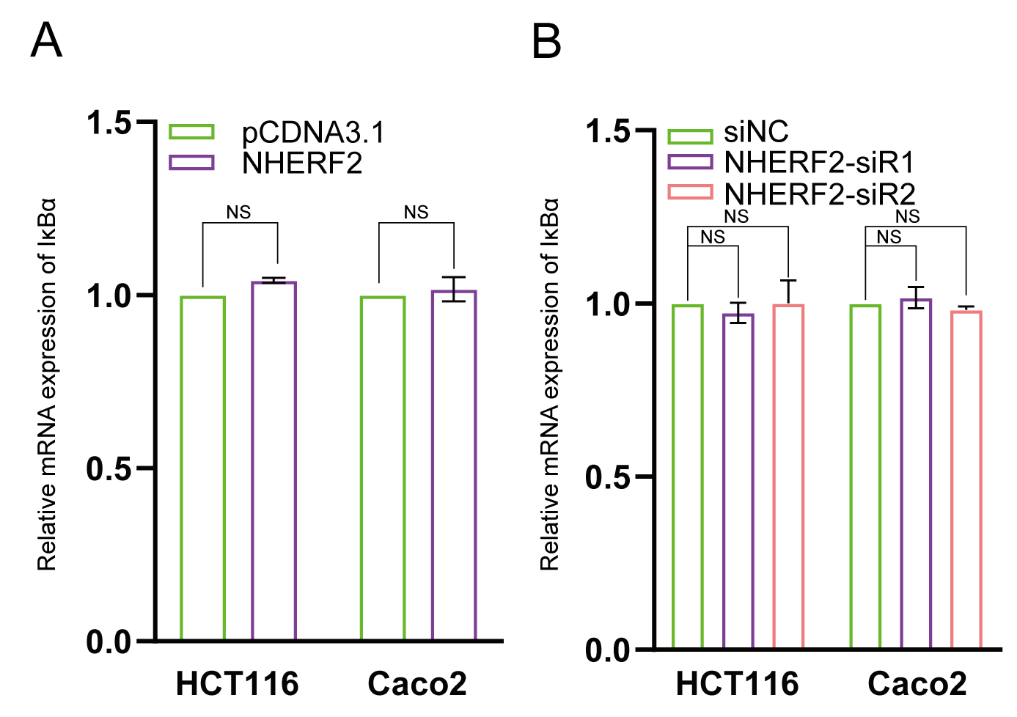


Fig.S5 The mRNA expression level of IκB. HCT116 and Caco2 cells were transfected with NHERF2 plasmid or pCDNA3.1 plasmid, the mRNA expression level of IκB was determined in the transfected cells by qPCR (A). HCT116 and Caco2 cells were transfected with NHERF2-siR1, or NHERF2-siR2, or siNC, mRNA expression level of IκB in the transfected cells by qPCR (B). Quantitative analyses of western blotting results were shown in the graphs. Data are presented as mean ± SD of three independent experiments. ns: not significant. *p < 0.05, **p < 0.01, ***p < 0.001 based on the Student’s t test.


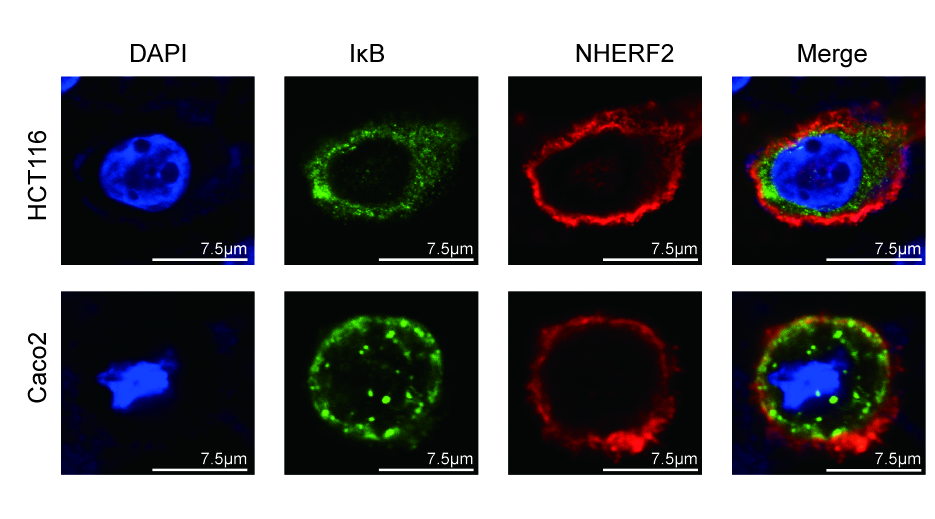


Fig.S6 Cellular colocalization of IκB with NHERF2. In HCT116 cells and Caco2 cells co-transfected with Flag- IκB and Myc-NHERF2. The nucleus was stained by DAPI (blue), Flag- IκB(green) and Myc-NHERF2(red) were observed by laser scanning confocal microscopy. Scale bar = 7.5 μM.


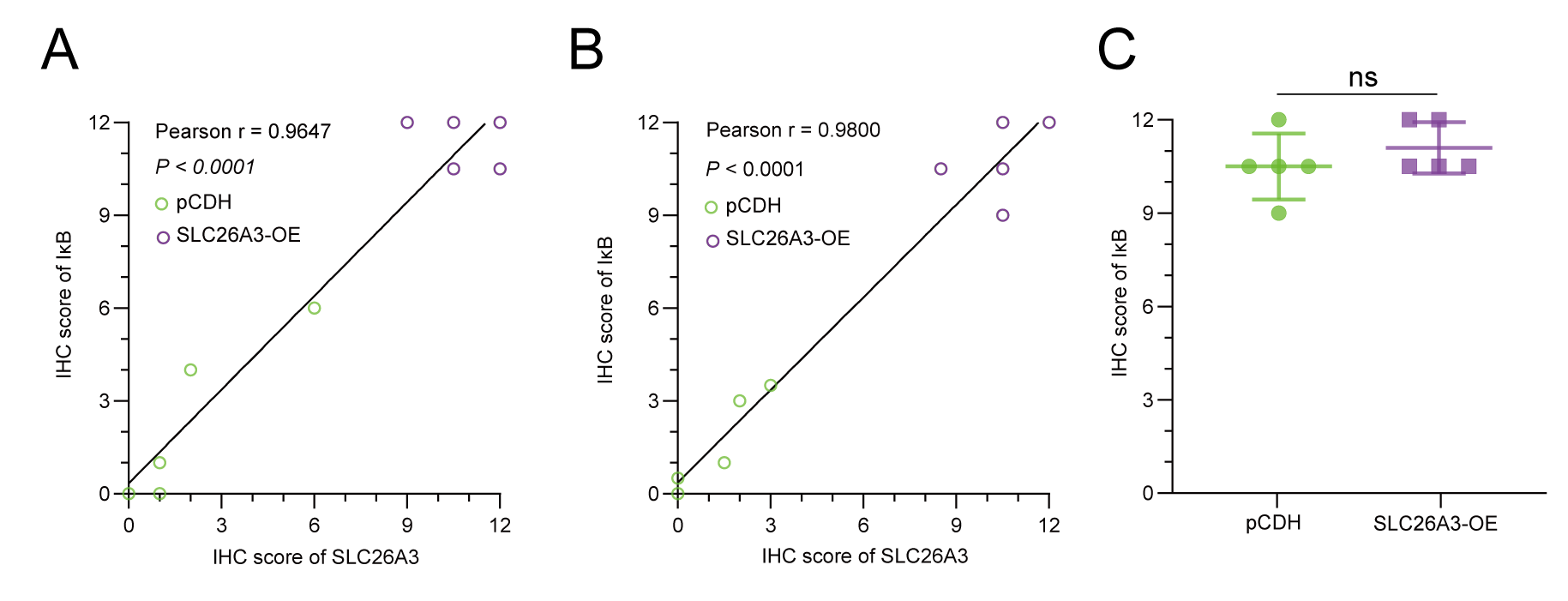


Fig.S7 Expression correlation between SLC26A3 and IκB in subcutaneous and orthotopic transplant tumors in mice. (A) Correlated protein expression level of SLC26A3 and IκB in subcutaneous transplant tumors in mice, comparing SLC26A3 overexpression group with the control group. (B) Correlated protein expression level of SLC26A3 and IκB in orthotopic transplant tumors in mice (solid tumor). (C) The protein expression level of IκB in orthotopic transplant tumors in mice (mucosa).

Table S1. Clinicopathological characteristics of 100 patients with CRC according to IHC score of SLC26A3

| Characteristics | Total | SLC26A3 expression | | *P*-value |
| --- | --- | --- | --- | --- |
|  |  | Low | High |  |
| Age (years) |  |  |  |  |
| <60 | 34 | 19 | 15 | 0.398 |
| ≥60 | 66 | 31 | 35 |  |
| Gender |  |  |  |  |
| Male | 62 | 33 | 29 | 0.410 |
| Female | 38 | 17 | 21 |  |
| Clinical stage |  |  |  |  |
| Stage I/II | 60 | 28 | 32 | 0.414 |
| Stage III/IV | 40 | 22 | 18 |  |
| Depth of tumor invasion |  |  |  |  |
| T1/2 | 28 | 18 | 10 | 0.075 |
| T3/4 | 72 | 32 | 40 |  |
| Lymph node metastasis |  |  |  |  |
| N0 | 54 | 22 | 32 | **0.045** |
| N+ | 46 | 28 | 18 |  |
| Distant metastasis |  |  |  |  |
| M0 | 93 | 44 | 49 | **0.050** |
| M1 | 7 | 6 | 1 |  |
| Differentiation |  |  |  |  |
| Low/undifferentiation | 46 | 29 | 17 | **0.016** |
| High/middle | 54 | 21 | 33 |  |
| Lymphovascular invasion |  |  |  |  |
| Absent | 52 | 27 | 25 | 0.689 |
| Present | 48 | 23 | 25 |  |
| Nerve invasion |  |  |  |  |
| Absent | 57 | 26 | 31 | 0.313 |
| Present | 43 | 24 | 19 |  |

Bold values indicate statistical significance with P < 0.05. Median IHC score for tumor tissues was 1.75. All patients were divided into two groups according to the result of their IHC score. The higher than 1.75 points are regarded as the high expression group, and the lower expression group is the opposite

**Table S2. Primer sequences and antibodies**

| **Target** | **Type** | **Sequence** |
| --- | --- | --- |
| Negative Control | siNC | TTCTCCGAACGTGTCACGT |
|  | shNC | TTCTCCGAACGTGTCACGT |
| SLC26A3 | qPCR probes | F: GCAGCAGGTGTATCCTACGG |
|  |  | R: TGCAAATGCAACCATTGCGA |
|  | pCDH | F:GATTCTAGAGCTAGCGAATTCGCCACCATGGATTACAAGGATGACGACGATAAGGGAGATTACAAGGATGACGACGATAAGATCGATTACAAGGATGACGACGATAAGATTGAACCCTTTGGGAATCA |
|  |  | R:ATCCTTCGCGGCCGCGGATCCTTAGAATTTTGTTTCAACTGGCACC |
|  | pCDNA3.1  full length | F:CTTGGTACCGAGCTCGGATCCATGGATTACAAGGATGACGACGATAAGGGAGATTACAAGGATGACGACGATAAGATCGATTACAAGGATGACGACGATAAGATTGAACCCTTTGGGAATCA |
|  |  | R:AACGGGCCCTCTAGACTCGAGTTAGAATTTTGTTTCAACTGGCACC |
|  | pCDNA3.1  1-524 | F:CTTGGTACCGAGCTCGGATCCGCCACCATGGATTACAAGGATGACGACGATAAGGGAGATTACAAGGATGACGACGATAAGATCGATTACAAGGATGACGACGATAAGATTGAACCCTTTGGGAATCA |
|  |  | R:AACGGGCCCTCTAGACTCGAGTTATTTTTTATTCTTATAGATGTTGGTTCTTC |
|  | pCDNA3.1  525-764 | F:CTTGGTACCGAGCTCGGATCCGCCACCATGGATTACAAGGATGACGACGATAAGGGAGATTACAAGGATGACGACGATAAGATCGATTACAAGGATGACGACGATAAGGATTATTATGATATGTATGAGCCAG |
|  |  | R:AACGGGCCCTCTAGACTCGAGTTAGAATTTTGTTTCAACTGGCACC |
| NHERF2 | qPCR probes | F: CAGATGAACACTTCAAGCGGC |
|  | pCDNA3.1  full length | R: CAGGTCACTTCGGGACGAG |
|  |  | R:AACGGGCCCTCTAGACTCGAGTCAGAAGTTGCTGAAGATTTCACG |
|  | pCDNA3.1  1-288 | F:CTTGGTACCGAGCTCGGATCCGCCACCATGGAGCAGAAACTCATCTCTGAAGAGGATCTGGCCGCGCCGGAGCCGCT |
|  |  | R:AACGGGCCCTCTAGACTCGAGTCACACGACCAGCAGCCG |
|  | pCDNA3.1  288-338 | F:CTTGGTACCGAGCTCGGATCCGCCACCATGGAGCAGAAACTCATCTCTGAAGAGGATCTGGACCCCGAGACAGATGAA |
|  |  | R:AACGGGCCCTCTAGACTCGAGTCAGAAGTTGCTGAAGATTTCACG |
|  | Si-1 | F: GGAACAGGAAGCGUGAAAUCUTT |
|  |  | R:AGAUUUCACGCUUCCUGUUCCTT |
|  | Si-2 | F: GGCAGAAUGUGGAGGGACUTT |
|  |  | R:AGUCCCUCCACAUUCUGCCTT |
|  | PEGX-4T-1 | F:GATCTGGTTCCGCGTGGATCCATGGCCGCGCCGGAGCCGCT |
|  |  | R:GTCACGATGCGGCCGCTCGAGTCAGAAGTTGCTGAAGATTTCACG |
| IκB | pCDNA3.1 | F:CTTGGTACCGAGCTCGGATCCGCCACCATGGATTACAAGGATGACGACGATAAGGGAGATTACAAGGATGACGACGATAAGATCGATTACAAGGATGACGACGATAAGTTCCAGGCGGCCGAG |
|  |  | R:AACGGGCCCTCTAGACTCGAGTCATAACGTCAGACGCTGGCC |
|  | qPCR probes | F:AAGTGATCCGCCAGGTGAAG |
|  |  | R:CTGCTCACAGGCAAGGTGTA |

The antibody

| **Name** | **Source** | **Catalog nunber** |
| --- | --- | --- |
| SLC26A3 | Santa Cruz | Sc-376187 |
| NHERF2 | Santa Cruz | Sc-365388 |
| IκB | CST | #4814 |
| p- IκB | CST | #2859 |
| NF-κB | CST | #8242 |
| p-NF-κB | CST | #3033 |
| GAPDH | CST | #5174 |
| Anti-Mouse | CST | #14709 |
| Anti-Rabbit | CST | #14708 |
| Goat Anti-Rabbit IgG H& L （Alexa Fluor 647） | Abcam | Ab150083 |
| Goat Anti-Mouse IgG H& L （Alexa Fluor 488） | Abcam | Ab150113 |
| Goat Anti-Rabbit IgG H& L （Alexa Fluor 488） | Abcam | Ab150077 |
| Goat Anti-Mouse IgG H& L （Alexa Fluor 647） | Abcam | Ab150115 |
| IκB | Abcam | Ab183134 (For IF) |
| 488-conjugated Myc tag | Proteintech | CL488-60003 |
| 594-conjugated HA tag | Proteintech | CL594-51064 |
| 647-conjugated DYKDDDDK tag | Proteintech | CL647-80010 |
